# Deep learning and remote photoplethysmography powered advancements in contactless physiological measurement

**DOI:** 10.3389/fbioe.2024.1420100

**Published:** 2024-07-17

**Authors:** Wei Chen, Zhe Yi, Lincoln Jian Rong Lim, Rebecca Qian Ru Lim, Aijie Zhang, Zhen Qian, Jiaxing Huang, Jia He, Bo Liu

**Affiliations:** ^1^ Department of Hand Surgery, Beijing Jishuitan Hospital, Capital Medical University, Beijing, China; ^2^ Department of Medical Imaging, Western Health, Footscray Hospital, Footscray, VIC, Australia; ^3^ Department of Surgery, The University of Melbourne, Melbourne, VIC, Australia; ^4^ Department of Hand & Reconstructive Microsurgery, Singapore General Hospital, Singapore, Singapore; ^5^ Institute of Intelligent Diagnostics, Beijing United-Imaging Research Institute of Intelligent Imaging, Beijing, China; ^6^ Institute of Automation, Chinese Academy of Sciences, Beijing, China; ^7^ School of Artificial Intelligence, University of Chinese Academy of Sciences, Beijing, China; ^8^ Beijing Research Institute of Traumatology and Orthopaedics, Beijing, China

**Keywords:** artificial intelligence, computer vision, rPPG, deep learning, physiological measurement

## Abstract

In recent decades, there has been ongoing development in the application of computer vision (CV) in the medical field. As conventional contact-based physiological measurement techniques often restrict a patient’s mobility in the clinical environment, the ability to achieve continuous, comfortable and convenient monitoring is thus a topic of interest to researchers. One type of CV application is remote imaging photoplethysmography (rPPG), which can predict vital signs using a video or image. While contactless physiological measurement techniques have an excellent application prospect, the lack of uniformity or standardization of contactless vital monitoring methods limits their application in remote healthcare/telehealth settings. Several methods have been developed to improve this limitation and solve the heterogeneity of video signals caused by movement, lighting, and equipment. The fundamental algorithms include traditional algorithms with optimization and developing deep learning (DL) algorithms. This article aims to provide an in-depth review of current Artificial Intelligence (AI) methods using CV and DL in contactless physiological measurement and a comprehensive summary of the latest development of contactless measurement techniques for skin perfusion, respiratory rate, blood oxygen saturation, heart rate, heart rate variability, and blood pressure.

## 1 Introduction

### 1.1 Computer vision

Computer Vision (CV) is a branch of science that studies how to make machines “see.” CV aims to generate a high-level understanding of the input images or videos, enabling computers to have similar levels of human perception and task execution. CV trains machines to perform these functions, but they rely on cameras, data, and algorithms to do their work in less time, unlike humans, who are dependent on the retina, optic nerve, and visual cortex (Aloimonos and Rosenfeld, 1991). CV is widely used in many industries, such as Medicine, Energy, Public Utilities, Manufacturing, and Automotive industries. A key factor driving the growth of these applications is the steady flow of visual information from smartphones, security systems, cameras, and other visual inspection devices. The rapid progress of CV over the past decade is primarily due to three factors: 1) the maturity of deep learning (DL), 2) strides in Graphic Processing Unit (GPU), and 3) the open sourcing of large, labeled datasets with which are used to train these algorithms (Esteva et al., 2021).

### 1.2 Remote imaging photoplethysmography

Photoplethysmography (PPG) is used to measure blood flow and evaluate the physiological status of patients. Its principle is based on the optical intensity change of reflected or transmitted light from a light source that passes through a microvascular tissue bed with pulsatile blood flow (Tamura, 2019). The PPG waveform signal contains two key components: 1) the alternating current (AC) component, which fluctuates with the change of blood volume between systole and diastole in the cardiac cycle, and 2) the direct current (DC) component, which corresponds to the optical signal transmitted or reflected from the tissue and is dependent on the tissue structure and the average arterial and venous blood volumes (Tamura, 2019). Based on this principle, PPG can represent physiological signs related to blood flow, such as heart rate, pulse, blood pressure, blood oxygen saturation, and skin perfusion. While PPG sensors have several advantages over ECG sensors (easy to use, low cost, convenient, etc.), direct skin contact is needed to restrict a patient’s movement. It also has limited application in patients with significant skin conditions (burns/ulcers/wounds) and immature skin (infants).

**FIGURE 1:**
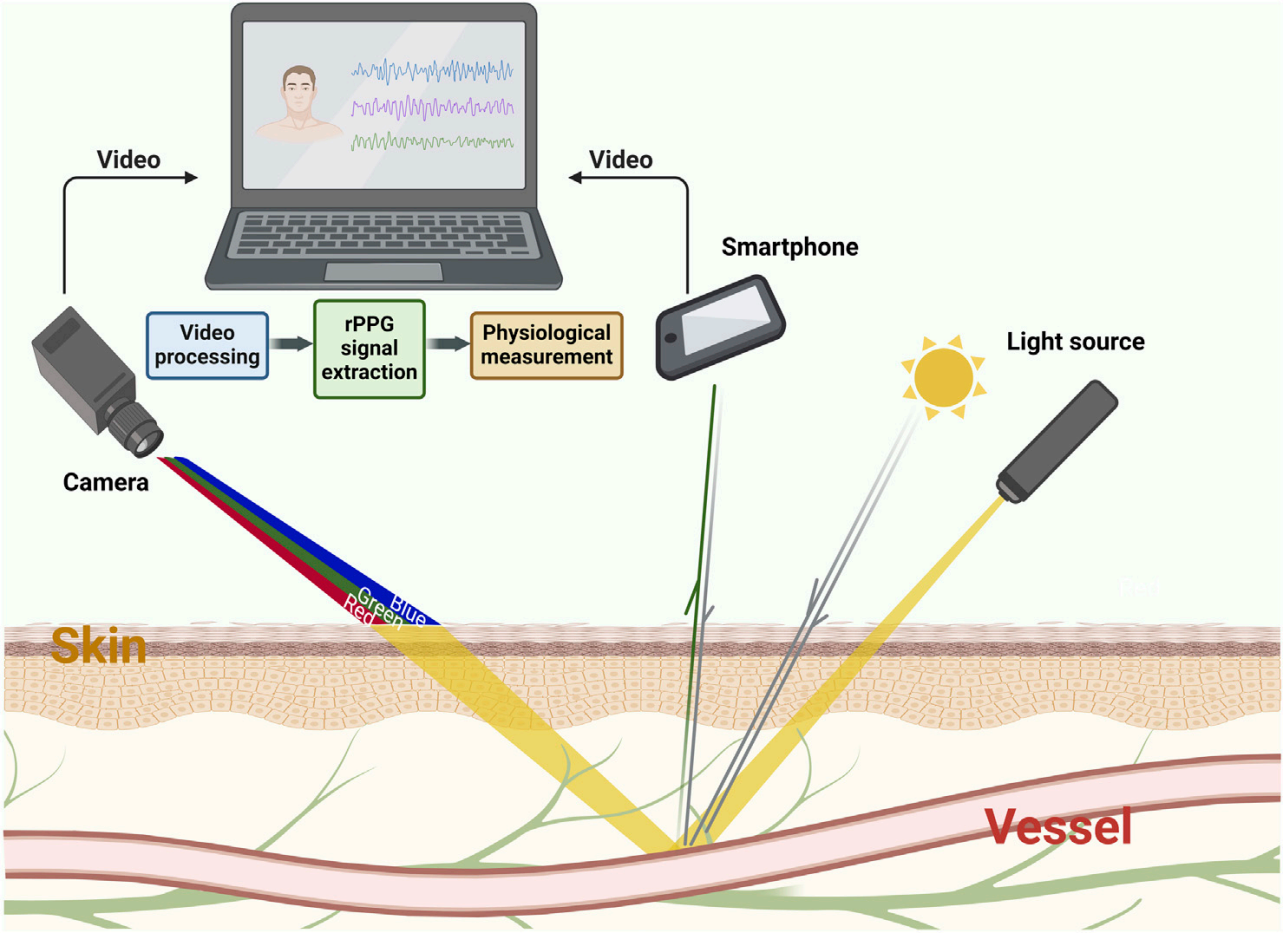
The schematic diagram of rPPG principle. The absorption and reflection of light by the skin varies according to the hemodynamic status under light sources, such as sunlight or lamps. Such changes will be recorded by imaging devices (including industrial cameras, webcams, cell phone lenses and other imaging devices) in the form of videos or pictures. Through the processing of computer and algorithm, rPPG waveforms that represent physiological information can be obtained from these videos.

As the application of CV in the field of healthcare, remote imaging photoplethysmography is a new technique based on the principle of PPG, which can sense the blood flow signal of outer skin layers (Marcinkevics et al., 2016). Compared to traditional contact PPG (cPPG), rPPG uses imaging devices (including industrial cameras, webcams, cell phone lenses, and other imaging devices) rather than a single sensor (e.g., photodiodes). This allows simultaneous assessment of multiple skin areas remotely. The skin’s absorption and reflection of light will change according to the patient’s hemodynamic status. Minor fluctuations of reflected light will carry specific physiological information, such as microcirculation perfusion, respiratory rate (RR), Oxygen saturation (SpO2), pulse rate (PR), heart rate (HR), and blood pressure (BP), etc., which can be read by traditional cameras (Jeong and Finkelstein, 2016; Gupta et al., 2020; Rasche et al., 2020; Lan et al., 2022; Boccignone et al., 2023). Figure 1 shows the schematic diagram of the rPPG principle. Presently, a research hotspot in the CV field is on achieving high-precision rPPG techniques in a low-cost and simplified way. The development and optimization of algorithms is one way to accomplish this goal. In search of the most robust algorithm for the extraction of the BVP signal from video recordings, numerous methods have been proposed: color-space-based [including red-green-blue (RGB), YCbCr, and hue-saturation-value (HSV)], blind-source-separation-based [BSS-based, including independent/principal component analysis (ICA and PCA), ensemble averaging (EA), empirical mode decomposition (EMD), and singular spectrum analysis (SSA)], model-based [including chrominance-based (CHROM), blood-volume-pulse-vector based (PBV), and plane-orthogonal-to-skin (POS)], and data-based [including spatial-subspace-rotation (2SR)] (Chaves-gonzalez et al., 2010; Poh et al., 2010; Sikdar et al., 2016; Wang et al., 2016; Wang et al., 2017a; Yu et al., 2021; Harford et al., 2022; Haugg et al., 2023). Table 1 summarizes these non-DL rPPG signal extraction methods.

**TABLE 1:** Summary of non-DL rPPG signal extraction methods.

	Non-DL rPPG method	Summary
Color-space-based	RGB	Based on the premise of high similarity between the green channel signal and the rPPG signal, the rPPG signal is characterized by the ratio of the green channel to the other two channels or sum, which can retain all the information of the RGB three channels (Haugg et al., 2023). However, only two distortions can be eliminated by linear combination of all three color channels which is usually not sufficient.
	YCbCr	Skin pixel clustering is performed on the Cb-Cr plane of the YCbCr color space, then the Cb and Cr components are expanded n times to generate a single BVP signal. This method can effectively reduce the signal distortion caused by the subtle change of skin color between the frames (Yu et al., 2021).
	HSV	Skin baseline hue and saturation vary depending on the skin and lighting colors, while HSV color model can best indicate skin color changes independent of ambient luminance (Chaves-gonzalez et al., 2010).
BSS-based	ICA	As a multi-dimensional statistic analysis method, independent component analysis can recover independent signals from a set of observations composed of linear or nonlinear mixtures of the underlying sources, such as BVP signals representing physiological measurements (Poh et al., 2011; Zhang et al., 2017; Favilla et al., 2019; Qi et al., 2019; Shi et al., 2023). However, the assumption that ICA depends on the independence of data components may lead to the inaccuracy of its application to components with correlation.
	PCA	The function of principal component analysis is to reduce dimensionality and extract major (i.e., of larger energy) and orthonormal components from signals or data (Lee et al., 2021; Alsheikhy et al., 2023; Lie et al., 2023). However, it may lose some important information, such as the respiratory signal hidden in the motion artifacts.
	EMD	Empirical mode decomposition is a powerful analytical tool used to effectively describe non-linear and non-stationary time series with rapidly varying frequencies (Lv et al., 2021; Shi et al., 2023). However, when there are intermittency caused by abnormal events (such as discontinuous signals, impulse interference and noise) in the signal, the wrong time-frequency distribution may be obtained, resulting in inaccurate results.
	SSA	Self-adaptive SSA is a global analysis method based on phase space reconstruction which decomposes original signal into multiple variable components and choose appropriate components automatically to reconstruct pulse wave according to singular value (Wang et al., 2020). However, how to determine the threshold to distinguish signal components from noise components according to singular values is a problem.
Model-based	CHROM	Chrominance-based signal processing method explicitly extract pulse signal against specular and motion artifacts. The RGB channels were projected into a chrominance subspace where the motion component was greatly eliminated (Song et al., 2020; Song et al., 2021; Zheng et al., 2022; Van et al., 2023).
	POS	POS defines a plane orthogonal to the temporally normalized skin-tone direction through using 2SR (a data-driven approach), so require less accurate knowledge of the BVP signature and are more tolerant to the amount of distortions, and the order in which it eliminates distortions is the opposite of CHROM (Shoushan et al., 2021; Zhang et al., 2023). CHROM is expected to be more vulnerable due to a (over time) consistent difference between the assumed and actual directions of the specular distortion for each individual, while POS is expected to be more vulnerable to inhomogeneous illumination spectra

Abbreviations: rPPG, imaging photoplethysmography; RGB, red-green-blue; ICA, independent component analysis; PCA, principal component analysis; EMD, empirical mode decomposition; SSA, singular spectrum analysis; CHROM, chrominance; POS, plane-orthogonal-to-skin; HSV, hue-saturation-value; BSS, blind-source-separation.

### 1.3 Deep learning

In recent years, the maturity and ongoing progress in the space of DL have injected new vitality into the CV field. DL-based CV techniques have been used in cardiology, pathology, dermatology, ophthalmology, and gastroenterology (Esteva et al., 2021). DL uses simple representations to extract abstract and higher-level features from data and uses artificial neurons as functional units to simulate human cognitive reasoning. The process of learning to perform tasks is called model training, and the ultimate goal of training is to minimize the error between the predicted results of the model and the ground truth. DL often involves three critical types of deep neural network (DNN): recurrent neural network (RNN), generative adversarial network (GAN), and convolutional neural network (CNN). At present, CNN is the most widely used CV. The structure of CNN is composed of three layers: 1) an input layer, 2) a hidden layer, and 3) an output layer. The process of CNN image classification usually includes dataset labeling, model learning, and performance evaluation (Helmy et al., 2023). This model can train a deeper network structure, extract more abstract image features, and reduce the number of neuron parameters to obtain better results with higher efficiency. DL has been successfully applied in contactless physiological and pathological measurements in recent years. Much has been achieved in the CV field, particularly in image registration, image retrieval, and image reconstruction and enhancement. With the support of the ever-increasing availability of datasets, DL will be pivotal in the rapid progress in medical image processing and analysis.

## 2 Peripheral blood perfusion

Changes in skin and flap color, temperature, or overall appearance (spots, swelling, etc.) often reflect a disease process. However, these changes are conventionally identified during clinical examination, which can be subjective and difficult to quantify. Digital cameras can provide an objective tool for real-time monitoring of skin changes, and this can be enhanced with rPPG signal analysis. Studies have shown that the amplitude of AC components in rPPG waveforms usually fluctuates with changes in central blood pressure or skin perfusion caused by local vasoconstriction (Tusman et al., 2019). The objective measurement of skin and flap blood perfusion can be achieved through the joint analysis of AC and DC components.

### 2.1 Skin perfusion

rPPG signals are affected by the wavelength of light, measurement site, motion artifacts, ambient light intensity, and ambient temperature (Tamura, 2019). Greenlight PPG signal can accurately reflect the change of skin blood flow caused by ambient temperature changes, while infrared light PPG signal does not reflect the change of skin blood flow under cold stimulation. Thus, skin blood perfusion information can be obtained using green light signals (Maeda et al., 2011). Furthermore, RGB color space is easily affected by luminance. By converting RGB pixel intensity values into the HSV color model, the interference of skin color changes related to ambient brightness can be eliminated (Chaves-gonzalez et al., 2010). Based on these, Harford et al. (2022) explored whether rPPG signals and color measurements could detect skin perfusion changes induced by drugs (phenylephrine and glyceryl trinitrate). They confirmed that skin perfusion changes induced by central (rather than local) administration could be detected from the rPPG waveforms of the skin. Similarly, rPPG signal intensity positively correlates with laser speckle imaging (LSI), used as a reference index for evaluating skin perfusion. This will enable practical evaluation of autonomic nervous system activity and skin perfusion (Rasche et al., 2020). In addition, using the rPPG positioning technique with a lock-in amplification algorithm and volumetric scan of the facial skin using a handheld swept-source optical coherence tomography (SS-OCT), the system can display the 3D structure of human skin microvasculature and obtain high-fidelity video of hemodynamic signals (He and Wang, 2022). The structural design of the exoscope combined with capillaroscopy and rPPG technique can reliably visualize the skin micro-vessels and study their local morphological characteristics. This can be used for the diagnosis and treatment of diseases related to blood microcirculation disorders (Machikhin et al., 2021). At the cellular level, vascular endothelial cells regulate vascular tension by releasing vasoactive substances such as nitric oxide and prostacyclin. As such, the changes of skin microcirculation perfusion caused by local heating detected by rPPG may also be extrapolated and used to evaluate endothelial function (Kamshilin et al., 2022a).

For different application scenarios, the imaging modalities and algorithms are different. Still, the fundamental purpose is to provide more auxiliary information for clinical diagnosis and treatment based on the detection of skin microcirculation. However, there are still some defects in the detection of skin microcirculation perfusion, such as local vascular disorders that will cause direct disturbance to the peripheral blood pulsation and contaminate the quantified measurements of microcirculation. In addition, the microcirculation situation may differ among individuals, and the algorithm’s applicability may need to be further optimized, such as the need for a large number of healthy datasets for correction or even considering additional imaging modalities to provide trans-regional calibration for microvascular measurements.

### 2.2 Flap perfusion

**FIGURE 2:**
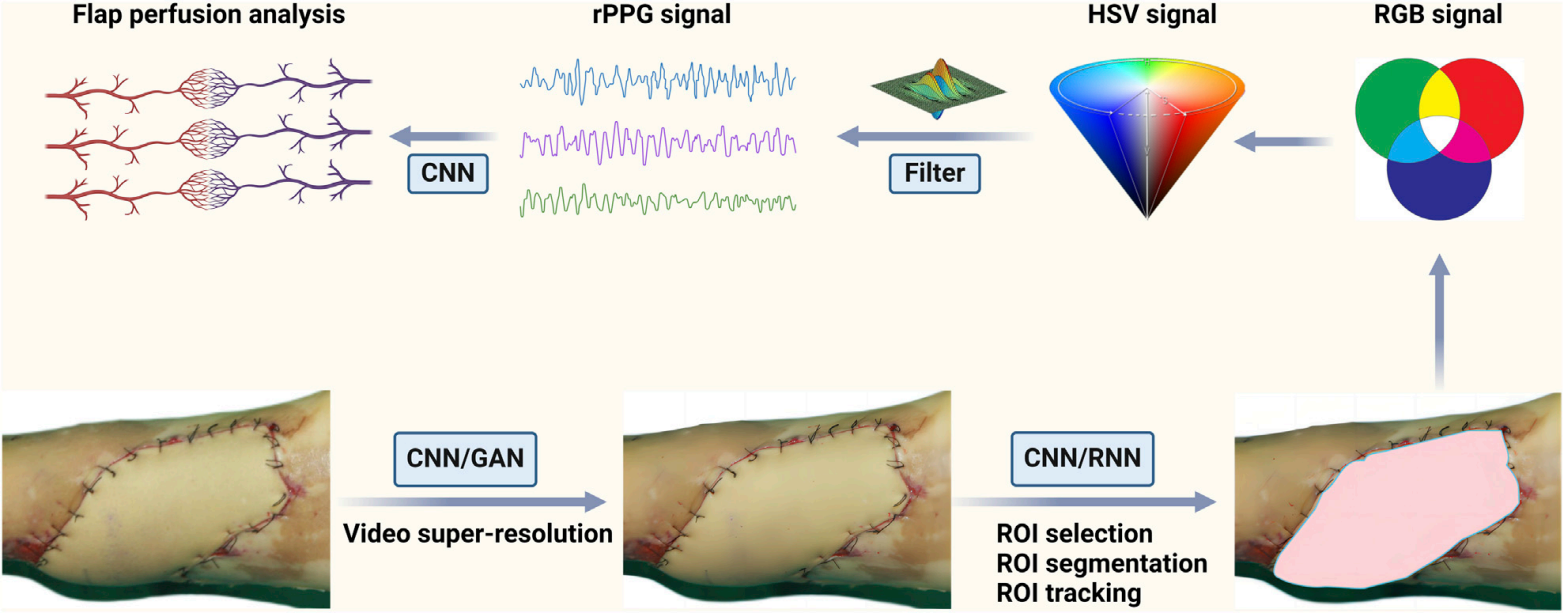
An ideal pipeline for contactless monitoring of flap blood supply. Firstly, the DL-based method is applied to original videos or images to realize super-resolution. Then the DL-based method is used to accomplish the preprocessing steps such as ROI selection, segmentation and tracking. Additionally, RGB signal can be converted into a HSV model with more detailed information. And the raw rPPG signal is obtained through a series of algorithms. Finally, the DL-based method is used to process the raw rPPG signal to obtain accurate physiological information.

rPPG technique also performs well in post operative tissue perfusion and wound evaluation (Zaunseder et al., 2018; Mamontov et al., 2020; Kamshilin et al., 2022b; Lai et al., 2022). A systematic review published in 2022 evaluated the performance of near-infrared spectroscopy (NIRS) and hyperspectral imaging (HSI) in testing for flap failure following reconstructive surgery (Lindelauf et al., 2022). While both techniques allow for non-invasive skin flap blood supply monitoring, each modality has limitations. NIRS monitoring of tissue blood oxygen is achieved through a contact sensor (non-aseptic). While continuous monitoring can be achieved, it is unsuitable for all flap types and intraoperative monitoring. On the other hand, HSI is a contactless method that monitors flap perfusion. This can be applied to the intraoperative monitoring of all flap types (e.g., fascio-cutaneous, muscle, intestinal). However, the main limitation is its insufficient real-time monitoring ability. This makes postoperative monitoring tedious and labor-intensive. In recent times, Schraven et al. (2023) achieved continuous analysis of local flap perfusion based on the rPPG technique. This study utilized high-resolution and fully digital surgical microscopy for imaging. It put forward three parameters for evaluating perfusion quality robustly: perfusion index, correlation coefficient of the analyzed rPPG signal with a reference rPPG signal (a reference skin region), and magnitude of the flap. This identified flaps with perfect post operative reperfusion, specific incidents (e.g., vasospasm) during reperfusion, and complete failure. This allowed for early, immediate anastomotic revision to prevent flap failure. This promising result solves the defect of NIRS contacting the flap and overcomes the limitation that HSI can not be continuously monitored. However, this study only explored the practical results of rPPG monitoring during surgical procedures. Postoperative monitoring is also crucial and more complicated in clinical practice. Several parameters are used to distinguish arterial crisis and venous crisis, including flap color, flap temperature, capillary refill time, and swelling degree of the flap. However, these parameters are not absolute, and the experience of microsurgery practitioners is more important. Therefore, establishing a multi-parameter DL model to identify flap crises and achieve early warning is a promising way to solve this clinical problem. Figure 2 shows an ideal pipeline for contactless monitoring of flap blood supply.

A Taiwan study developed a smartphone application called “How’s the Flap” based on Apple’s CoreML framework for early flap crisis warning (Hsu et al., 2023). The datasets of this study contain internal training (230 cases of normal vs. 34 cases of congestion) and external validation (240 cases of normal vs. 16 cases of congestion), including 840 photographs of flaps with varying backgrounds, illumination intensity, flap sizes, and shapes. The accuracy of the model’s training and validation datasets reached 0.922 and 0.923, respectively. Finally, the Application was used to analyze 921 photographs to distinguish flap congestion, and the accuracy was 0.953. Although this study trains a satisfactory model, it is only suitable for venous crises that are easy to detect in clinical practice, and it may be more important to identify critical arterial crises. Therefore, the random forest ML model was proposed to identify arterial and venous insufficiency from images (Huang et al., 2023). The model was trained (80%) and validated (20%) using 805 flap photographs of 176 patients (555 cases of normal, 97 cases of arterial insufficiency, 153 cases of venous congestion), and Shapley Additive Explanations (SHAP) was used to explain the model. The results showed that the temperature and RGB values of flap color could predict the arterial and venous crises, respectively, and the model’s accuracy was 0.984. However, the photographs were segmented to enlarge datasets, which may lead to less generalizability and high homogeneity of the algorithm. In addition, the proposed model’s robustness is worth discussing because the flap photographs were taken in a standardized environment (the same background and illumination intensity).

## 3 Respiratory rate and oxygen saturation

### 3.1 Respiratory rate

RR is a vital sign that aids in detecting and evaluating respiratory dysfunction. Conventional electrocardiography (ECG) sensors and respiration belts are reliable methods for monitoring RR. The change of respiratory-induced rPPG waveform is usually related to the effect of respiration on cardiac activity, namely, respiratory-induced variation (RIV). The effect of respiration on the intensity of BVP, amplitude of cardiac output, and HR will enable rPPG waveforms to be used to measure RR (Buda et al., 1979).

#### 3.1.1 Conventional methods for contactless RR estimation

Two main kinds of approaches have been proposed in the literature to achieve contactless RR estimation: 1) methods based on the direct extraction of morphological features attributable to breathing (that is, RIV) (Scully et al., 2012; Nam et al., 2014; Lázaro et al., 2015; Charlton et al., 2018) and 2) methods aimed at isolating the motion trend due to HR and RR (Wei et al., 2017; Schrumpf et al., 2019), implicitly related to RIV. For the first method, incremental merge segmenting (IMS) is the most utilized method. It uses several solutions to fuse the morphological features of respiration (Karlen et al., 2015). The second method is the most promising, single-channel BSS-based method to separate RR from HR and noise. The EMD and SSA are commonly used methods (Huang et al., 1998; Boccignone et al., 2023). Research shows that the morphological estimation of RIV is more reliable than those produced by a single-channel BSS-based method (Boccignone et al., 2023). However, a BSS-based method based on the selected dual region of interest (ROI) developed by Wei et al. (2017) obtained facial BVP and the respiratory signals corresponding to respiratory motion artifacts, thus achieving contactless synchronous measurement of RR and HR. Unlike other studies that rely on sophisticated video tracking and detection algorithms to attenuate motion artifacts, this algorithm takes advantage of motion artifacts and obtains hidden respiratory signals. Extension and improvement of this method may have the potential to detect multiple physiological indicators at the same time.

Unlike visible and near-infrared imaging systems, infrared thermography (IRT) does not require additional lighting and can work in a completely dark environment. For people who need to monitor asleep breathing (e.g., people with a substantial risk of sleep apnea) and critically ill patients who often wear oxygen masks, the rPPG signal provided by IRT may be a way of contactless monitoring of RR (Li et al., 2014a; Chan et al., 2019; Zhu et al., 2019). The skin of infants is fragile and sensitive to light stimulation. It is also challenging to use their small noses as an anatomical marker. In this instance, IRT based on a “black box” algorithm is a viable choice to evaluate RR (Pereira et al., 2019). However, the robustness of these algorithms’ development based on ward and family scenarios may not perform well. In complex public settings, the robust breath-tracking method based on the mobile thermal imaging system proposed by Cho et al. (2017) counteracts the confounding effects of ambient temperature changes and motion artifacts. This would enable accurate RR assessment in highly dynamic thermal scenes.

#### 3.1.2 DL model for contactless RR estimation

Hardware improvements only provide limited gains in non-contact-based measurement accuracy. DL is a way to achieve high-precision rPPG technique in a simple and low-cost approach and is a current research hotspot in the CV field. The DL algorithm with CNN may achieve the purpose of extracting accurate rPPG signals from low-quality videos. BlazeFace and FaceMesh are face detection models based on MobileNetV1/V2 architecture, which can accurately locate the ROI (Bayar et al., 2022; Maity et al., 2022; Jewel et al., 2023; Kolosov et al., 2023). Accurate remote contactless RR estimation can be achieved with Eulerian Video Magnification (EVM) and rPPG techniques (Kolosov et al., 2023). However, the throughput, power consumption, efficiency, and value (throughput/cost) may differ when it runs on different commercial off-the-shelf hardware platforms. In addition, a multi-task temporal shift convolutional attention network (MTTS-CAN) also achieves contactless vital measurements and predicts both rPPG and respiratory signals (Liu et al., 2020). However, it will require complicated preprocessing. The Multi-task Siamese (MTS) model proposed by Lee et al. (2022a) combines the advantages of the Siamese neural network (based on 3D CNN) and multi-task architecture. This reduced the number of parameters by 16 times and accurately predicted heart and respiratory signals in a facial-based video. The MTS model outperformed the single-task model as well as the conventional multitask learning model for RR estimation, was computationally lightweight and may be helpful for applications in smartphones or portable devices. As mentioned, thermal imaging has many advantages and is one of the essential means to achieve contactless RR detection. However, due to the lack of information, selecting and tracking ROI in neonatal thermal images is challenging. One way around this is using the YOLO5Face (based on CSPNet) detection model to recognize the ROI in an RGB image and register it to thermal imaging. This can effectively solve the problem of extracting RR from neonatal thermal photos (Maurya et al., 2022). Whether based on the motion signal and rPPG signal in RGB video or the respiratory signal in thermal imaging video, the DL model can be trained through rich datasets to realize the dynamic estimation of RR. The only thing we need to do is to continuously simplify the algorithms and achieve robust RR estimation in the future.

### 3.2 Oxygen saturation

SpO_2_, the relative concentration of oxygenated hemoglobin relative to total hemoglobin, is one of the vital physiological indicators commonly used to monitor a patient’s respiratory function. The traditional finger-type photoelectric sensor is inconvenient for patients requiring long-term continuous essential monitoring. With rPPG techniques, remote pulse oximetry (RPO) can help with contactless vital monitoring. The principle that RPO can assist in SpO_2_ evaluation is based on the ratio of AC/DC ratios between two wavelengths of interest proposed by Beer-Lambert ‘s law. The limitation of this law is that it only considers the absorbance of chromophores in skin tissue and ignores the existence of light scatter (Kocsis et al., 2006). The robustness of RPO is also related to camera performance, light wavelength, motion artifacts, ambient light intensity, individual differences, posture, and temperature (Wieringa et al., 2005; Humphreys et al., 2007; Kong et al., 2013; Shao et al., 2016; Moço et al., 2019; Moço and Verkruysse, 2020). For visible light, different wavelengths penetrate the skin at different depths. The rPPG signals obtained by using blue and green wavelengths as light sources come from the arterioles of the upper dermal layers, while the signals received by using red wavelengths come from subdermal tissue (Verkruysse et al., 2017). This depth-gap may be more apparent when the skin properties or physiological conditions change (e.g., posture and temperature changes), so the robustness of visible light-based RPO in detecting SpO_2_ may be reduced (Moço et al., 2019; Moço and Verkruysse, 2020). However, these factors that affect the robustness of RPO are challenging to solve simultaneously, whether the improvement of equipment or algorithm is more aimed at a particular factor.

#### 3.2.1 Multi-spectrum for enhancing RPO robustness

Applying a multi-wavelength light source or multi-spectral camera can effectively reduce the decline of RPO accuracy caused by the changes in ambient light. Wieringa et al. (2005) verified the feasibility of applying a three-wavelength light source to RPO measurement for the first time, but this method has not been well applied due to low SNR. Although the joint use of a dual-wavelength light-emitting diode array and semiconductor camera can estimate SpO_2_ measurements, its acquisition frame rate is low and is not highly accurate (Humphreys et al., 2007). The CMOS camera with trigger control function alternately records the lip rPPG signals of two specific wavelengths. This has the best SNR under orange and near-infrared wavelengths combined illumination. However, the accuracy of this method is still dependent on many surrounding environmental factors (Shao et al., 2016). While Kong et al. (2013) achieved accurate SpO_2_ measurement in ambient light using two cameras with narrowband filters to capture rPPG signals at two different wavelengths; the required equipment is complex and will not be readily applicable in the clinical setting.

Dynamic spectrum (DS) has the advantage of suppressing individual differences and measurement conditions. Li et al. (2014b) applied this theory to extract DS from the frequency domain of rPPG signals to calculate SpO_2_. The multispectral camera plays a significant role in material composition detection based on spectral imaging and can achieve fast and contactless material detection and recognition. Lan et al. (2022) used the multi-spectral camera to obtain the multi-wavelength rPPG signal from facial video, extract the DS values of multiple wavelengths, and obtain SpO_2_ measurements. This method simultaneously solves the influence of ambient illumination and individual differences on rPPG signals. It can potentially meet the needs of contactless SpO_2_ detection in a convenient and fast way. To further improve the robustness of RPO when detecting SpO_2_ under visible light sources, some calibration methods based on skin color, posture, and temperature changes have been proposed (Guazzi et al., 2015; Moço et al., 2019; Moço and Verkruysse, 2020).

#### 3.2.2 Smartphone used for RPO

However, multispectral-based devices (light sources and cameras) are often inconvenient, expensive, and complex. The development of RPO based on smartphones has thus attracted more attention. Smartphones can record and analyze the varying color signals of a fingertip placed in contact with its optical sensor and can effectively evaluate HR, RR, and SpO_2_ (Scully et al., 2012; Nam et al., 2014; Karlen et al., 2015; Lázaro et al., 2015). Previous studies have successfully used rPPG signals from smartphones to estimate SpO_2_ (based on the traditional Beer-Lambert law) and introduced a multiple linear regression (MLR) algorithm to calibrate the RPO robustness decline caused by changes in physiological conditions (Sun et al., 2021). For special populations (e.g., children), this method has also been proven to monitor RR and intrathoracic pressure. It can also assist in diagnosing pneumonia and stratification of its severity (Lucy et al., 2021). Although the SpO_2_ detection based on Smartphones generally reflects peripheral tissue SpO_2_ and can not simulate the arterial SpO_2_ provided by the contact pulse oximetry, the mobile device with the built-in color camera as a remote sensor and flashlight as illumination is simple and readily available. With rapid advancements in smartphone technology, more opportunities for medical applications will arise. This will help to improve access to medical technology in undeveloped areas, as well as telehealth care and home health monitoring. Therefore, integrating accurate RR and RPO monitoring techniques into smartphones will accelerate the development of telehealth.

## 4 Heart rate/pulse rate

Cardiovascular pulse can be estimated and finally used in PR and HR estimation by analyzing the temporal signals of micro-motion or color variations across time. Studies have shown that for consumer cameras (e.g., a webcam or mobile camera), facial video is more reliable for evaluating HR than other body parts (such as wrists and calves) (Wang C. et al., 2018; Van der kooij and Naber, 2019). In the past decade, numerous studies have been conducted on HR detection through rPPG signals provided by facial video. There is currently a wide variety of model designs, parameter settings, algorithms, and equipment. Several methods have been developed for HR estimation using dimensionality reduction (e.g., BSS-based method), optical modeling (e.g., green channel), motion-based methods, and machine learning (ML). These methods are usually applied to face video processing, face BVP signal extraction, and HR computation phases to achieve HR detection (Wang C. et al., 2018).

**FIGURE 3:**
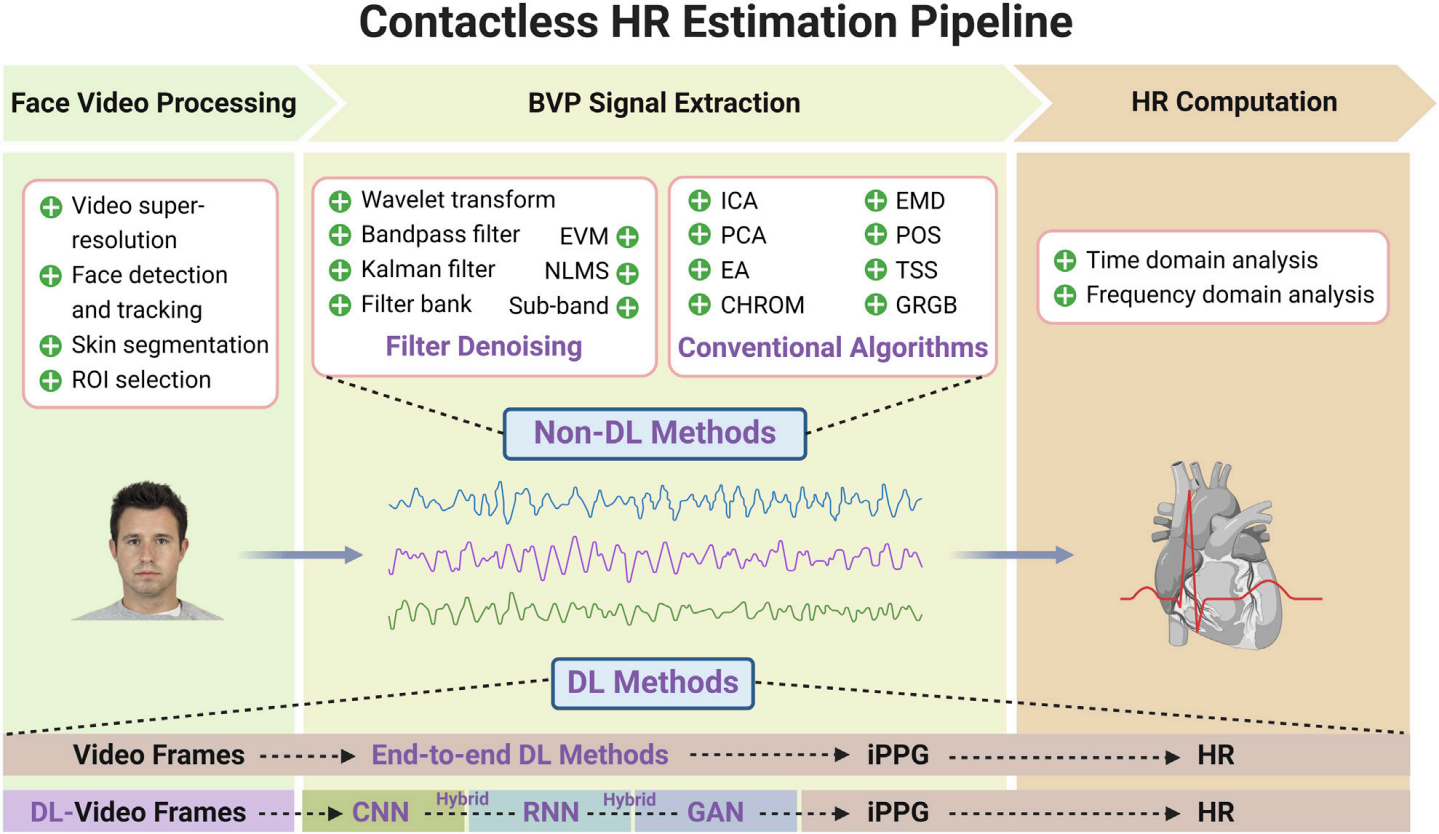
Contactless HR estimation pipeline based on videos. The contactless HR estimation pipeline is composed of face video processing, face BVP signal extraction and HR computation. Face video processing includes video super-resolution, face detection and tracking, skin segmentation, and ROI selection. BVP signal extraction includes the filter denoising methods for motion artifacts filtering and skin color normalization and the conventional algorithms for raw BVP signals construction. HR computation methods are divided into time domain analysis and frequency domain analysis. DL algorithms can be divided into end-to-end type and hybrid type. The former directly establish the mapping from video frames to the target HR values or BVP signals, while the latter use DL model in conjunction with traditional ML methods or different DL models to deal with different stages of HR estimation. The face image in the schematic diagram comes from Chicago Face Database (Ma et al., 2015).

Face video processing includes face detection and tracking, skin segmentation, and ROI selection. These processes aim to detect the face, improve the motion robustness, reduce the quantization error, and prepare the feature signal for further BVP signal extraction (Huang and Dung, 2016; Gudi et al., 2020; He et al., 2021; Woyczyk et al., 2021). However, some scholars have proposed a method of extracting HR from the whole video by ignoring the ROI selection and tracking process. However, this method is only suitable for instances with a stable video background environment over time (for example, sleep monitoring) (Wang W. et al., 2018). BVP signal extraction includes several postprocessing methods, such as bandpass filtering, detrending, and wavelet transform. This improves the accuracy of HR estimation by cleaning, filtering, or denoise rPPG signal (Huang and Dung, 2016; He et al., 2021). HR computation methods are divided into time domain analysis (peak detection methods) and frequency domain analysis (Malik et al., 1996; Sun et al., 2012). Some studies have tried to put forward unsupervised clustering-based methods to replace traditional peak detection, but they are still not as accurate as the improved BVP signal extraction method (Lee et al., 2019). A system review published in 2018 concluded that a facial skin area extraction, ICA, and peak detection pipeline achieved state-of-the-art accuracy (Wang C. et al., 2018). With the development of CV, these methods are being optimized and, at times, used to complement each other. As subtle facial color changes caused by cardiovascular activity are affected by noise such as ambient light, facial expressions, breathing, camera parameters, out-of-plane movements, and unconscious head shaking, researchers in the field of CV are mainly interested in how to reduce the interference of external factors and how to extract BVP signals quickly and accurately. Figure 3 shows the contactless HR estimation pipeline based on videos (including three phases).

### 4.1 Denoising for face video signal processing

#### 4.1.1 Motion artifacts filtering

Motion artifacts are the most common interference factor in video recordings. A considerable number of methods have been developed to reduce or eliminate the error caused by motion artifacts, including Sub-band rPPG, continuous wavelet transform, bounded Kalman filter technique, and motion index (MI) indicator (Wang et al., 2017b; Lin et al., 2017; Finžgar and Podržaj, 2018; Prakash and Tucker, 2018; Abdulrahaman, 2023). The extent of eliminating the noise signals from the pulse signal in rPPG depends on the dimensionality of the acquired video signal. The Sub-band rPPG method proposed by Wang et al. (2017b) not only processes the given RGB signal in high dimension but also suppresses the distortion signals of each component, which effectively improves the robustness of multi-wavelength rPPG. Furthermore, the continuous wavelet transform-based Sub-Band rPPG method (SB-CWT) increases the degrees of freedom of distortion elimination by exerting wavelet transform decomposition on RGB video signals (Finžgar and Podržaj, 2018). This method has a good SNR and can estimate PR from RGB video signals without significant motion scenes. In addition, combined with a blur identification and denoising algorithm for each frame and a bounded Kalman filter technique for motion estimation and feature tracking, motion artifacts such as blur and noise caused by head motion can be minimized, but its application in complex and widely moving scenes needs further research (Prakash and Tucker, 2018). Lin et al. (2017) designed a motion index (MI) indicator to filter motion artifacts and used complexion tracking to detect the moving state of the target. At the same time, the near-infrared camera could achieve a better dark mode measurement of PR but ignore the diversity of complexion between individuals. The wavelet transform involves a two-stage denoising method proposed by Abdulrahman (2023), effectively removes motion artifacts, can significantly enhance the reconstructed signal, and can be applied to HR video monitoring of natural motion (not quick or large motions) scenes at different times of the day. Therefore, for different motion scenes, the demand for the algorithm to filter motion artifacts may be different. Additionally, the potential effects of varying skin colors caused by complexion or light source must be considered.

#### 4.1.2 Skin color normalization or enhancement

In addition to motion artifacts, skin color is a crucial factor affecting the robustness of the rPPG signal. The skin color is affected by the change in light source and complexion, which brings much noise to the acquisition of the rPPG signal. The anti-interference performance of the normalized least mean square (NLMS) adaptive filter can rectify the illuminance variation. Still, it needs the desired signal established by a smooth rectifier in the background as the input, which is difficult to realize (Li et al., 2014c). A Distance-PPG method based on filter banks can weigh the average skin color changes in different tracking regions of the face and has an excellent anti-noise performance. Still, the algorithm implemented by this method is complex and time-consuming, and the pulse wave extracted by this method can not see apparent dicrotic waves (Kumar et al., 2015). Based on these limitations, Wang et al. (2020) first removed baseline offset and high-frequency random noise. Then, they used a self-adaptive SSA algorithm to extract details-preserving pulse waves from facial video in real situations.

Color enhancement can magnify subtle skin color changes. Unlike the traditional video based on RGB color space, the video based on YCbCr color space can obtain more subtle skin color changes, thus realizing the accurate extraction of BVP signals (Yu et al., 2021). Microsoft Kinect (a multi-mode camera) can provide additional information for RGB data, namely, depth, infrared, and skeleton frames, and processes the RGB images through the EVM color augmentation method to magnify the skin color changes caused by blood flow, so it is developed as a contactless HR estimation technique (Gambi et al., 2017). By integrating denoising techniques such as amplitude selective filter (MASF), wavelet decomposition, and robust PCA on RealSense (an RGB-NIR dual-modality camera), depth information can be obtained from short videos and HR information can be obtained accurately (Lie et al., 2023). Furthermore, Martinez-Delgado et al. (2022) combined a face detection algorithm based on OpenCV with the EVM algorithm to achieve a more accurate HR estimation. In addition, the EVM video amplification technique is usually used in combination with the DL model or PCA algorithm in HR estimation (Kolosov et al., 2023; Lin et al., 2023).

These video signal enhancement methods for filtering motion artifacts and dealing with skin color changes are the prerequisites for accurate rPPG signal extraction. However, rPPG signals often need further processing to obtain the components of BVP signals for accurate HR measurement. This step usually involves many more advanced algorithms, such as ICA and CHROM.

### 4.2 Conventional algorithms for contactless PR/HR estimation

#### 4.2.1 Single ICA

As a commonly used method for BVP signal extraction, ICA begins with a random initialization of unmixing matrix with just a single prerequisite of unmixing matrix dimension, depending on the number of independent components, which is comparatively trivial than the wavelet transform method. ICA algorithm regards BVP extraction as a BSS problem, that is, extracting the desired signal with no or limited information from the mixed signal. Algorithms such as joint diagonalization approximation of matrices (JADE) and FastICA, which show motion tolerance to some extent, are based on the transformation or improvement of ICA (Poh et al., 2011; Shi et al., 2023). In addition, the multi-channel ICA algorithm is based on second-order blind identification (SOBI), which was proposed by Zhang et al. (2017) realizes the possibility of evaluating HR under low illumination. Similarly, integrating multiple simultaneously acquired BVP signals extracted by the ICA algorithm can also measure HR reliably (Favilla et al., 2019). The “Project_ICA” algorithm uses the skin reflection model to extract the BVP signal from the facial rPPG signal (Qi et al., 2019). This method combines advanced techniques such as feature point detection tracking and skin pixel detection, overcomes the decrease in robustness caused by motion artifacts and weak light and dark skin, and performs better than several classical ICA, CHROM, 2SR, and POS algorithms. However, it still has significant limitations in the application of black skin. Different algorithms have different advantages; for example, the ICA algorithm can recover independent signals from mixed signals, the CHROM algorithm explicitly extracts pulse signals against specular and motion artifacts, and the EMD is a powerful analytical tool used to effectively describe non-linear and non-stationary time series with rapidly varying frequencies. The high complexity of algorithms usually requires a longer running time, and how to combine the advantages of different algorithms to achieve fast and accurate HR estimation is a topic that scholars are committed to discussing.

#### 4.2.2 Hybrid ICA

As one of the most commonly used and practical conventional algorithms, the ICA algorithm is often used with other algorithms to predict HR. Song et al. (2020) combined the advantages of ICA in independence and CHROM (a model-based method) in dealing with chromaticity, proposed a Semi-BSS-based rPPG method to realize the best performance of HR estimation. Still, this method requires super-high resolution (2.7 k) video. Combined with the remote ballistocardiography (rBCG) technique, rPPG signals can realize the combination of color and motion of BSS-based (EA, PCA, and ICA), thus effectively reducing the impact of illumination changes and motion artifacts on HR evaluation (Lee et al., 2021). In 2021, Lv et al. (2021) proposed an improved ensemble EMD (EEMD) algorithm, namely, complete EEMD with adaptive noise (CEEMDAN), and combined it with FastICA to realize remote HR measurement. However, there is still residual white noise in CEEMDAN, which leads to decomposition errors. To ensure the elimination of noise, the number of iterations of the algorithm will increase, which will lead to an increase in time cost. To solve the problem of decomposition errors and slow running speed caused by this residual noise, Shi et al. (2023) improved both EEMD and FastICA algorithms. By adding zero-mean random white noise generated according to the input signal to the sampled data, the Huber derivative approximation function is used instead of the nonlinear function in the FastICA algorithm to improve further accuracy, robustness, timeliness, and anti-interference performance. In addition, an under-complete ICA algorithm was proposed to restrict motion and illumination variation artifacts (Gupta et al., 2022). By using a non-linear cumulative density function (CDF) optimized by customized Levenberg-Marquardt algorithm (LMA) to estimate the unmixing matrix, this method can retain all the information of RGB three channels and has an excellent performance in constrained motion and illumination variations scenarios.

#### 4.2.3 Other algorithms

Color subspace transformation methods such as CHROM and POS use orthonormal vector transformations to construct raw signals for BVP extraction (Wang et al., 2017a). Compared with the conventional ICA algorithm, it does not lose the critical information in the red and blue channels. Still, its main disadvantage is that improper weights assigned to color channels may reduce the BVP information (Gupta et al., 2022). POS algorithm can not only extract high-precision PR from videos captured by high-speed cameras but also process BVP signals in multiple respiratory modes (spontaneous, metronome, and forced) and video (smartphone and webcam) under different types of body movements, but it is challenging to achieve synchronization or desynchronization between HR and RR cycles (Shoushan et al., 2021; Zhang et al., 2023). A self-adaptive SSA algorithm can obtain cyclical components, remove aperiodic irregular noise, and extract the pulse wave that keeps the details from the facial video in real situations (Wang et al., 2020). The T-SNE-based signal separation (TSS) method can decompose the observed color traces into pulse-related vectors and noise vectors and then select the vector with the most significant spectral peak as the BVP signal for HR measurement (Wang et al., 2022). This proposed method is suitable for RGB and HSV color spaces and significantly suppresses the noise caused by head movement. Still, it is not robust to complex light interference and violent sports interference scenes. However, without relying on complex mathematical models or ML algorithms, combining RGB channels alone may also be a way to obtain robust BVP signals. Research shows that the sum of the green-to-red channel and green-to-blue channel ratios (GRGB) not only has lower computational complexity but also has the same effect as the POS algorithm, especially suitable for videos with a lot of movements and indoor lighting (e.g., gym and rotation) (Haugg et al., 2023). Table 2 summarizes these conventional rPPG signal extraction algorithms for HR estimation. Although there are many mature methods of using CV techniques based on traditional algorithms to extract rPPG signals used to estimate HR, the decline in the robustness of HR evaluation caused by subject motion and ambient lighting variations can still be optimized. Due to the success of DL in many CV and medical image processing applications, DL methods have been considered for rPPG to deal with its challenges.

**TABLE 2:** Several conventional iPPG signal extraction algorithms for HR measurement.

ROI selection	Feature type	Proposed algorithms	Correlation coefficient (compared to pulse oximeter)	Basic architecture	HR computation methods	Measurement
Center 60% width of the face	RGB	JADE (Poh et al., 2011)	1.00, 0.92 (HR, HRV)	ICA	A custom algorithm to obtain IBI	HR, HRV
Area containing eyes	RGB	SOBI (Zhang et al., 2017)	>0.90	ICA	Time-domain kurtosis	HR
Forehead, cheek	Single channel 560 ± 20 nm	FastICA (Favilla et al., 2019)	≥0.999, ≥0.998 (HR, HRV)	ICA	A multi-scale algorithm for peak estimation	HR, HRV
Face skin area	RGB	Project_ICA (Qi et al., 2019)	0.76, 0.74, 0.69, 0.47 (stationary, interaction with computer, swinging heads, exercise recovery)	ICA	FFT for peak estimation	HR
Cheek	RGB	CHROM, KDICA (Song et al., 2020)	0.981 vs. 0.968, 0.918 (CHROM alone, KDICA alone)	CHROM, ICA	FFT for peak estimation	HR
Face skin area	RGB	CDF optimizing LMA, undercomplete ICA (Gupta et al., 2022)	0.92, 0.94, 0.92 (constrained, motion, illumination variations scenarios)	LMA, ICA	FFT for peak estimation	HR
Forehead, cheek, nose	RGB	ZCA (Iozzia et al., 2016)	0.961 vs. 0.927, 0.911 (CHROM, ChromICA)	PCA	FFT, AR time series modeling	PRV
Forehead, cheek	Green channel	Modified EEMD, modified FastICA (Shi et al., 2023)	0.85 vs. 0.39, 0.35, 0.75, 0.55, 0.78 (EEMD, ICA, FastICA, EEMD + FastICA, CEEMDAN + FastICA)	EMD, ICA	FFT for peak estimation	HR
Forehead, cheeks, whole face	RGB	— (Zhang et al., 2023)	0.945	POS	FT for peak estimation	PR
Around mouse and nose	Green channel	TSS (Wang et al., 2022)	0.95 vs. 0.76, 0.74, 0.84, 0.88, 0.93 (ICA, CHROM, POS, SSA, rPPGNet)	T-SNE	Temporal filtering and FT	HR
Forehead, cheek	RGB	GRGB (Haugg et al., 2023)	CRGB > GR > GB	G-R ratio, G-B ratio	FFT for peak estimation	HR
Face exclude forehead	RGB	— (Van et al., 2023)	0.994, 0.971 (POS, CHROM) vs. 0.583, 0.916 (PCA, ICA)	CHROM, POS	IBI	PRV
Face skin area	RGB	PVM with GEVD (LI et al., 2020)	<0.90	PCA, PVM	FFT for peak estimation	PRV
Face skin area	Green channel	self-adaptive SSA (Wang et al., 2020)	0.91	SSA	Peak estimation	HR

Abbreviations: ICA, independent component analysis; CHROM, chrominance; JADE, joint diagonalization approximation of matrices; SOBI, second-order blind identification; KDICA, kernel density ICA; CDF, cumulative density function; LMA, Levenberg-Marquardt algorithm; ZCA, zero-phase component analysis; PCA, principal component analysis; EMD, empirical mode decomposition; EEMD, ensemble EMD; POS, plane-orthogonal-to-skin; T-SNE, T-distributed stochastic neighbor embedding; TSS, T-SNE-based signal separation; GRGB, the sum of green-to-red channel and green-to-blue channel ratios; PVM, periodic variance maximization; GEVD, generalized eigenvalue decomposition; SSA, singular spectrum analysis; FT, fourier transform; FFT, fast Fourier transform; AR, autoregressive; IBI, interbeat intervals.

### 4.3 DL for contactless PR/HR estimation

Before the advent of DL, several ML methods were used to remotely estimate HR, including linear regression, k-nearest neighbor (kNN) classifier, support-vector regression, adaptive hidden Markov models, and a general-to-specific transfer learning strategy named SynRhythm (Hsu et al., 2014; Monkaresi et al., 2014; Fan et al., 2015; Niu et al., 2018). As with many CV and signal processing applications, DL methods have shown promise in mapping complex physiological processes for contactless HR measurement. The number of research papers utilizing DL methods for remote HR measurement has increased yearly and is expected to grow continuously. The rPPG approaches for HR estimation based on DL can be generally divided into two types: 1) the end-to-end type and 2) the hybrid type. The former provides spatial-temporal (ST) visualization of physiological signals via the attention mechanism and directly establishes the mapping from video frames to the target HR values or BVP signals. At the same time, the latter uses the DL model in conjunction with traditional ML methods or DL models to deal with different stages of HR estimation (Figure 3).

#### 4.3.1 End-to-end DL model

A method is classified as end-to-end if it takes in a series of video frames as input and directly outputs the rPPG signal or HR without any intermediate steps (Figure 3). End-to-end DL methods are indisputably great tools due to their straightforward model optimization process.

##### 4.3.1.1 Single-stage CNN model

The Single-stage CNN model utilizes only one CNN architecture to extract HR or rPPG signals directly from facial video, even if there is no need for the preprocessing stage of face detection and tracking (Bousefsaf et al., 2019). The robustness of HR measurement under different skin types, facial expressions, and movements can be improved by integrating different attention mechanisms in CNN structure, such as motion, appearance, and ST attention model (Hu et al., 2022; Mcduff et al., 2022; Ouzar et al., 2023). An end-to-end ST network, X-iPPGNet, based on modified Xception integrated with a depthwise separable convolution, can realize instantaneous PR estimation directly from facial video recordings (Ouzar et al., 2023). Unlike most existing systems, X-iPPGNet has advantages with high and sharply fluctuating PR, ensuring robust PR prediction under various conditions (including head motions, facial expressions, and skin tone). This is because it learns the rPPG concept from scratch without incorporating prior knowledge or going through the extraction of BVP signals.

##### 4.3.1.2 Multi-stage CNN model

The Multi-stage CNN model utilizes two or more linear CNN architectures to achieve more than one phase of HR estimation. A two-stage CNN named HR-CNN composed of the Extractor and HR estimator is trained end-to-end through alternating optimization and is robust to illumination changes and subject motion (Spetlik et al., 2018). Unlike the commonly used COHFACE and MAHNOB databases, the datasets used for training in this study are a new open-source ECG-Fitness database whose videos are not compressed. Similarly, another two-stage 3D CNN method comprised of ST video enhancement network (STVEN) and rPPGNet (composed of an ST convolutional network, a skin-based attention module, and a partition constraint module) generalizes well on novel data with only compressed videos available, which implies the promising potential for real-world applications (Yu et al., 2019a). In addition, the end-to-end model proposed by Perepelkina et al. (2020) uses CNN architectures in the three stages of the HR estimation pipeline. After using RetinaNet (based on MobileNet) to process facial ROI, HeartTrack (based on a 3D ST attention CNN) obtained the time series. Finally, 1D CNN was used to calculate HR. Furthermore, an utterly self-supervised training method based on pre-trained ResNet18 and 3D PhysNet CNN was designed to get rid of expensive ground truth physiological training data (Gideon and Stent, 2021).

##### 4.3.1.3 Multi-scale network

We define a multi-scale network as a phase of HR estimation that uses more than one DL architecture; that is, the three-phase linear structure of the HR estimation pipeline is extended by multi-scale DL architecture. The Siamese-rPPG network proposed by Tsou et al. (2020) contains two 3D CNN architectures, which can not only extract the rPPG signal from the two face ROIs (without preprocessing) simultaneously but also effectively retain the ST characteristics of the rPPG signal. Furthermore, multi-task Siamese (MTS) combines the advantages of Siamese neural network and multi-task architecture to accurately predict cardiac signals while significantly reducing parameters (Lee et al., 2022a). Li et al. (2022) proposed a short-time end-to-end HR estimation framework based on facial features and temporal relationships of video frames. In the proposed method, a deep 3D multi-scale network with cross-layer residual structure is designed to construct an autoencoder and extract robust rPPG features by transferring the lost information in scale transformation. Then, an ST fusion mechanism is proposed to help the network focus on features related to rPPG signals. Yin et al. (2022) proposed an end-to-end multi-task learning model named PulseNet, combining the advantages of signal-based methods and DL methods, which can achieve accurate HR estimation in scenes that include changes in lighting and head movement. PulseNet uses (2 + 1)D convolution to decouple ST information and a skin-based attention mechanism to suppress background noise.

The central difference convolution (CDC) operator has potential advantages for rPPG feature extraction due to its ability to enrich temporal context. The 3D CDC network can achieve accurate HR measurement by combining the attention mechanism of ST, motion, and appearance, for example, the proposed CDCA-rPPGNet and AutoHR (Yu et al., 2020; Zhao et al., 2021; Liu et al., 2022). AutoHR proposed by Yu et al. (2020) is composed of neural architecture search (NAS) and the 3D temporal difference convolution (TDC). By combining a hybrid loss function considering constraints from both time and frequency domains and ST data augmentation strategies, AutoHR realizes accurate HR measurement. More complicatedly and accurately, a 3D ST convolutional network with multi-hierarchical fusion, including low-level face feature generation (LFFG), 3D ST stack convolution (STSC), multi-hierarchical feature fusion (MHFF), and signal predictor (SP), can reconstruct the rPPG signal representing HR from facial RGB video (Li et al., 2023).

##### 4.3.1.4 Transformer

Transformer, a recently developed DL model, differs from the convolution structure of CNN based on local connection and weight sharing. It is based on self-attention mechanisms (Vaswani et al., 2017). Although the structure of the Transformer model is complex and requires many parameters, it can handle data noise and deformation better than the CNN structure. Yu et al. (2022) first proposed an end-to-end video transformer architecture, PhysFormer, for remote physiological measurement. On the one hand, the cascaded temporal difference Transformer blocks in PhysFormer benefit the rPPG feature enhancement via global ST attention based on the fine-grained temporal skin color differences. On the other hand, to alleviate the interference-induced overfitting issue and complement the weak temporal supervision signals, elaborate supervision in the frequency domain is designed, which helps PhysFormer learn more intrinsic rPPG-aware features. To better exploit the temporal contextual and periodic rPPG clues, the PhysFormer was extended to the two-pathway SlowFast-based PhysFormer++ with temporal difference periodic and cross-attention Transformers (Yu et al., 2023). However, the application of the Transformer to the physiological measurement of rPPG is still in its infancy, and future research should focus on designing a more efficient architecture while exploring a more accurate and efficient ST self-attention mechanism, particularly for long-sequence rPPG monitoring. Table 3 summarizes the application of end-to-end DL methods in contactless HR estimation.

**TABLE 3:** Application of end-to-end DL methods in contactless HR estimation.

	Publication year	DL name/components	Attention mechanism	ST feature	Data preprocessing	Learning rate	Batch size	Loss function	Datasets	Measurement	Advantages
Single-stage CNN	2019	3D CNN	No	No	Synthetic rPPG video generator approach including five steps	10^–3^	25	CCE	UBFC-rPPG	PR	Training procedure employs only synthetic data without any special image preprocessing
	2020	3D CNN	No	Yes	Downsampleing and adding random uniform noises for data augmentation	—	1	MAE	MAHNOB-HCI, Fraunhofer Driver Emotion Study Dataset	HRV	Suitable for affective sensing
	2022	CAN	Motion and appearance	No	Changing subsurface color and subsurface scattering for synthesizing videos with pulse signals	—	36	MSE	AFRL, MMSE-HR, UBFC-rPPG	HR	Improve robustness to skin type and motion
	2022	2D and 3D CNN	ST	Yes	MTCNN to detect and crop coarse face areas and remove background	10^–2^ on PURE, 5 × 10^−4^ on COHFACE	128	NegPCC	PURE, COHFACE	HR	Reduce highly redundant spatial information and motion noise
	2023	X-iPPGNet	No	Yes	Performing face segmentation for eliminating the background and non-skin areas	10^–6^	128	MSE	BP4D+, MAHNOB-HCI, MMSE-HR, UBFC-rPPG	PR	No need to extract IPPG signal, no prior knowledge; fast convergence speed, and low computational cost
Multi-stage CNN	2018	HR-CNN	No	No	—	10^–4^ on extractor, 10^–1^ on HR estimator	128	MAE	COHFACE, MAHNOB, PURE, ECG-fitness	HR	Propose a robust uncompressed video database
	2019	STVEN, rPPGNet	Skin	Yes	Viola-Jones face detector to detect and crop coarse face areas and remove background	10^–4^	128, 64	NegPCC	OBF, MAHNOB-HCI	HR, HRV	Especially suitable for highly compressed videos
	2020	HeartTrack	ST	Yes	Increasing videos’ fps with intermediate frame synthesis	3 × 10^−5^	32	MSE	UBFC-rPPG, MoLi-ppg-1, MoLi-ppg-2	HR	Creat real-life MoLi-ppg-1 and MoLi-ppg-2 datasets
	2021	ResNet18, PhysNet	No	Yes	Estimating a bounding box around the face and adding an 50% scale buffer to the box for ensuring relative stability of the video	10^–5^	4	MCC	PURE, COHFACE, MR-NIRP-Car, UBFC-rPPG	HR	Fully self-supervised training, no reliance on ground truth data
Multi-scale Network	2019	PhysNet	No	Yes	Viola-Jones face detector to crop the face area at the first frame and fix the region through the following frames	10^–4^	2	NegPCC	OBF, MAHNOB-HCI	HR, HRV	First work to use deep ST networks for reconstructing precise rPPG signals
	2020	Siamese-rPPG Network	No	Yes	No	10^–4^	2	NegPCC	UBFC-rPPG, PURE, COHFACE	HR	Simultaneously learn different features from two facial regions
	2020	AutoHR	No	Yes	No	—	2	NegPCC and SNR	VIPL-HR, MAHNOB-HCI, MMSE-HR	HR	Find out three key factors (i.e., network architecture, loss function and data augmentation strategy) influencing the robustness and generalization ability of end-to-end rPPG networks heavily
	2021	3DCDC-T	Motion and appearance	Yes	No	5 × 10^−2^	12	Huber loss	UBFC-rPPG, PURE, COHFACE	HR, RR	Avoid pre-processing steps; enhance spatiotemporal representation with rich temporal context
	2022	MTS	Channel and spatial	Yes	Dlib to extract cheek and forehead for fixing the input size	10^–4^	1	PCC	COHFACE	HR, RR	Simultaneously predict HR and RR; a small number of parameters
	2022	3D residual CNN	ST	Yes	A face detector to locate the rough face position and a skin segmentation algorithm to preserve the skin region for better removal of background interference	10^–4^	8	Branching loss	OBF, COHFACE, UBFC-rPPG, PHY-100	HR	Construct a new dataset PHY-100; require fewer video frames
	2022	PulseNet	Skin	Yes	Dlib to detect faces and downsample and a segmentation algorithm to generate a binary face mask as the ground truth of skin segmentation	10^–4^	64, 128	MAE	UBFC-rPPG, PURE, COHFACE, MAHNOB-HCI	HR	Combine skin segmentation and attention mechanisms to suppress background noise; mutual constraints between iPPG signals and average HR values
	2022	CDCA-rPPGNet	ST	Yes	OpenFace to get facial landmarks	2 × 10^−4^	8	NegPCC	UBFC-rPPG, PURE	HR	Better capture the subtle color changes
	2022	ESA-rPPGNet	Spatial and channel	Yes	MTCNN for face detection	10^–3^	8	NegPCC	UBFC-rPPG, PURE	HRV	Effectively capture interchannel dependencies and pixel-level dependencies; greatly reduce the time complexity of the network
	2023	LFFG, STSC, MHFF, SP	Skin and motion	Yes	Cascading the ensemble of regression trees (ERT) algorithm to approximately crop the redundant background of the videos	10^–4^	32	NegPCC	UBFC-rPPG, COHFACE, FaceBio-v1	HR	Strengthen the ST correlation of multi-channel features; construct a new dataset FaceBio-v1
	2023	3D CNN, BiLSTM	Region	Yes	Video re-sampling to generate positive and negative samples	10^–5^	4	Series of frequency-inspired losses	UBFC-rPPG, PURE, DEAP, MMVS, BP4D+	HR, HRV, RR	A novel frequency-inspired self-supervised framework without the need of ground truth
Transformer	2022	PhysFormer	ST	Yes	MTCNN face detector to crop the enlarged face area at the first frame and fix the region through the following frames	10^–4^	4	Curriculum learning guided dynamic loss	VIPL-HR, MAHNOB-HCI, MMSE-HR, OBF	HR, HRV, RR	Recognize fine-grained temporal skin color differences; alleviate the overfitting issue; complement the weak temporal supervision signals
	2023	PhysFormer++	ST, cross- and self-attention	Yes	MTCNN face detector to crop the enlarged face area at the first frame and fix the region through the following frames	10^–4^	4	Curriculum learning guided dynamic loss	VIPL-HR, MAHNOB-HCI, MMSE-HR, OBF	HR, HRV, RR	Better exploit the temporal contextual and periodic rPPG clues

Abbreviations: DL, deep learning; HR, heart rate; HRV, heart rate variability; RR, respiratory rate; ST, spatio-temporal; CAN, convolutional attention network; CNN, convolutional neural network; STVEN, spatio-temporal video enhancement network; MTS, multitask Siamese; LFFG, low-level face feature generation; STSC, spatio-temporal stack convolution; MHFF, multi-hierarchical feature fusion; SP, signal predictor; CCE, categorical cross-entropy; MAE, mean absolute error; MSE, mean squared error; NegPearson, negative Pearson correlation coefficient; PCC, pearson correlation coefficient; MCC, maximum cross-correlation; SNR, signal-to-noise ratio.

Although the end-to-end DL model shows great potential in HR estimation, it often results in a mysterious black box model that is difficult to understand. Therefore, optimizing the algorithm based on various factors that affect the robustness of rPPG is necessary. In addition, multiple DL models applied at different stages of HR measurement may increase the interpretability of the process.

#### 4.3.2 Hybrid DL model

HR estimation is classified into three phases: face video processing, face BVP signal extraction, and HR computation. Using DL model(s) in one phase or different DL models in various phases is defined as hybrid DL, while the other phases still use the non-DL algorithms (Figure 3).

##### 4.3.2.1 DL for face video processing

BlazeFace is a face detection model based on MobileNetV1/V2 architecture developed by Google, while FaceMesh integrates a face landmark model based on BlazeFace. These two models can eliminate any facial redundant areas that have no impact on HR or RR estimation to accurately locate an ROI (Bayar et al., 2022; Maity et al., 2022; Pagano et al., 2022; Jewel et al., 2023; Kolosov et al., 2023; Odinaev et al., 2023). The proposed cascade residual CNN-FPNR technique used for preprocessing and SNR enhancement facilitates segmentation in low-light ambient videos and provides high frame quality for HR estimation (Gupta et al., 2023). The AND-rPPG method based on a 2D temporal convolution network (TCN) architecture enables denoise temporal signals and action units from facial videos (Lokendra and Puneet, 2022). Then, the denoised temporal signals from all the facial regions are consolidated to compute the rPPG signal and estimate the HR. As a component of a two-stage DL model, rPPGRNet based on recurrent back projection network (RBPN) can form super-resolution images and then be used for HR estimation of subsequent THRNet (based on 3D ResNet-10) (Yue et al., 2021). The proposed DeepMag based on CNN architecture enables automated magnification of subtle color and motion signals from a specific source, even in the presence of large motions of various velocities (Chen and Mcduff, 2020). The magnified videos produced by DeepMag have fewer artifacts and blurring than the traditional EVM method.

##### 4.3.2.2 CNN for face BVP signal extraction/feature decoder

A depth-wise separable convolution based on 3D MobileNet enables an estimate of HR from the feature images formed by spatial decomposition and temporal filtering of EVM (Qiu et al., 2019). Similarly, the proposed cross-verified feature disentangling strategy (CVD, based on CNN) enables disentangling the physiological features with non-physiological representations existing in a multi-scale ST map, which realizes robust multi-task physiological measurements (Niu et al., 2020a). In addition, a DL model based on ResNet-18 architecture is used to judge the quality of the ST feature image extracted by the conventional CHROM algorithm and to determine whether it is used in the fast Fourier transform (FFT) of subsequent HR estimation (Zheng et al., 2022). Similarly, for the ST images or time-frequency representation extracted by traditional algorithms, the CNN model can achieve robust HR estimation in continuous motion scenes (Hsu et al., 2017; Jaiswal and Meenpal, 2022; Chen and Li, 2023). Chen and Li (2023) applied CNN model based on ResNet101 architecture to HR reality monitoring in aerobics training with high accuracy. Jaiswal and Meenpal (2022) proposed a video-based noise-less cardiopulmonary measurement, which converts the 3D videos into 2D ST Images by wavelet decomposition, suppressing the noise while preserving temporal information of the rPPG signal. ST images are provided as input to CNN, which enables mapping the corresponding HR values under heterogeneous lighting conditions and continuous motion. Similarly, short-time Fourier transform (STFT) can transform the 1D color signal and frequency signal extracted from RGB videos to 2D time-frequency representation, subsequently used to train a VGG15 DL network to estimate the pulse (Hsu et al., 2017).

Temporal and spatial features are the key to accurately extracting rPPG signals from facial video. In addition to processing the ST signals obtained by traditional methods, CNN itself can also integrate the ST modular to improve the anti-noise ability, which is often realized by added attention mechanism or convolution modular, for example, the proposed DeeprPPG and ETA-rPPGNet networks (Niu et al., 2019; Liu and Yuen, 2020; Hu et al., 2021). Niu et al. (2019) input the ST map extracted from the video into the ResNet-18 CNN architecture integrated with channel attention and ST attention mechanism, thus outputting robust HR estimation. DeeprPPG, as a lightweight rPPG estimation network without preprocessing, is based on ST ConvNets (full 3D convolution/spatial 2D convolution + temporal 1D convolution), allows flexible ROI selection with different locations and sizes, and obtains the robust rPPG signal from multiple input skin regions (Liu and Yuen, 2020). The ETA-rPPGNet proposed by Hu et al. (2021) is comprised of a time-domain segment subnet and backbone net. The feature maps of the video generated by the time-domain segment subnet can effectively reduce redundant information. At the same time, the integrated time-domain attention mechanism in the backbone net can significantly improve the model’s anti-noise (insufficient light conditions and head movement) ability. ETA-rPPGNet shows superior performance in compressed datasets (compared with DeepPhys). Still, its short-term estimation performance is not as good as that of EVM-CNN because it needs to deal with redundant information. Furthermore, a novel global-local interaction and supervision network (GLISNet) utilizes the local path to learn the representations in the original scale and the global path to learn the representations in the other scale, thus capturing multi-scale information (Zhao et al., 2023). GLISNet can extract and fuse pulse signals from multi-scale ROIs without heavy computational load and preserve the rich temporal features of rPPG video to achieve accurate HR estimation.

##### 4.3.2.3 RNN (+CNN) for face BVP signal extraction/feature decoder

Long short-term memory (LSTM), a typical RNN architecture, enables filter rPPG signal obtained by conventional methods (POS, PCA, CHROM, CWT, etc.), can more accurately identify the changes of HR and further evaluate the mental state or physical function of the population (Slapnicar et al., 2019). A two-layer LSTM was designed for regression from raw signals after normalization to estimate pulse wave signals and generate a large scale of synthetic HR signals which is used to pre-train the LSTM network to prevent over-fitting (Bian et al., 2019). This algorithm can effectively alleviate the problem of insufficient HR public database and achieve better performance than the baseline method (GREEN, ICA, CHROM, and POS). Maity et al. (2022) proposed a bi-directional LSTM (Bi-LSTM) network to filter the motion distortions in the rPPG signals, which shows better-filtering capability over the discriminative signature-based filtering during HR estimation.

Combined with CNN architecture, LSTM may realize more advanced performance. The proposed HR evaluation method named Meta-rPPG was comprised of ResNet (2D CNN) for feature extraction and an LSTM network for rPPG estimation, whose performance of HR estimation was better than that of EVM in different datasets (Lee et al., 2020; Pagano et al., 2022). As the most common CNN architectures, U-Net or ResNet combined with LSTM outperform the widely used prior-knowledge rPPG methodology in PR estimation, for example, a combination of POS and CWT (Niu et al., 2020b; Lampier et al., 2022). Furthermore, the combination of AlexNet, ResNet50V2, and LSTM can extract HR information from the rPPG signal obtained by the PCA algorithm (Alsheikhy et al., 2023).

##### 4.3.2.4 GAN for face BVP signal extraction/feature decoder

GAN-based pulse feature disentanglement network (PFDNet) can extract the common robust features of rPPG and PPG pulse signals, and further recognize atrial fibrillation from facial videos with typical facial motions (Liu et al., 2023). The cbPPGGAN framework based on CycleGAN was used to enhance raw pulse signals extracted using traditional approaches while estimating more accurate HR under illumination variation (Yang et al., 2023). Furthermore, the proposed Dual-GAN model uses two GAN models to learn the mapping from the ST map to BVP and simulate noise distribution, respectively (Lu et al., 2021). The Dual-GAN structure allowed for indirect supervision for noise distribution and achieved better feature disentanglement for the BVP signal. This resulted in better prediction performance for HR, HRV, and RR. Table 4 summarizes the application of hybrid DL methods in contactless HR estimation.

**TABLE 4:** Application of hybrid DL methods in contactless HR estimation.

	Publication year	DL name	Backbone network	Attention mechanism	ST feature	Data preprocessing	Learning rate	Batch size	Loss function	Datasets	Measurement	Advantages
DL for video processing	2020	DeepMag	2D CNN	—	—	—	—	—	—	Dataset from Estepp et al.	HR, RR	Enable automated magnification of subtle color and motion signals
	2021	rPPGRNet	RBPN	—	—	—	10^–4^	8	MAE	MMVS, DEAP	HR	Recover the rPPG information during video super-resolution processing
	2022	AND-rPPG	2D TCN	—	—	—	10^–3^	—	SNR	UBFC-rPPG, COHFACE	HR	Mitigate the facial expression-based noise from the temporal signa
	2023	BlazeFace, FaceMesh	MobileNetV1/V2	—	—	Format the incoming frame to be compatible with the target CNN requirements	—	—	—	—	HR, RR	Eliminate any redundant areas of the face that do not contribute to HR or RR estimation
	2023	ICNet	Cascade CNN	—	—	EVM using the adaptive filter to raise the frame rate and SNR	10^–2^	—	—	UBFC-rPPG	HR, RR	Achieve strong segmentation in low-light ambient videos
CNN for BVP signal extraction	2017	—	15-layered VGG	No	Time-frequency	The second order derivatives, the detrend filtering, and the chrominance filtering to enhance the averaged ROI signal	10^–2^	64	—	PURE, MAHNOB-HCI	HR	Pioneering CNN framework for real-time pulse estimation; developed a pulse database called the Pulse from Face (PFF)
	2019	EVM-CNN	3D MobileNet	No	Yes	A regression local-binary-features-based approach for face detection and tracking	—	—	Euclidean distance	MMSE-HR	HR	HR is directly estimated from a feature image obtained by using spatial decomposition and temporal filtering
	2019	—	ResNet-18	Channel and ST	Yes	Downsampling for data augmentation	1.5 × 10^−3^	100	MAE	VIPL-HR, MMSE-HR	HR	Places focus on the salient features included in rPPG-signals
	2020	CVD	3D CNN	No	Yes	SeetaFace to detect the facial landmarks	5 × 10^−4^	—	MAE, PCC	VIPL-HR, OBF, MMSE-HR	HR, HRV, RR	Disentangle the physiological features with non-physiological representations; realize robust multi-task physiological measurements
	2020	DeeprPPG	Full 3D CNN or spatial 2D CNN + temporal 1D CNN	No	Yes	ROI video clips pre-obtained	2 × 10^−4^	64	NegPCC	PURE, COHFACE, MAHNOB-HCI	HR	Suitable for unseen skin regions and unseen scenarios
	2021	THRNet	3D ResNet-10	Temporalwise	Yes	rPPGRNet for super-resolution processing and rPPG information recovery	10^–4^	32	MSE	MMVS, DEAP	HR	Solve the key rPPG information loss problem
	2021	ETA-rPPGNet	3D cascade CNN	Time-domain	Yes	Locating the ROI area and segmenting skin to overcome the noise	5 × 10^−3^	—	NegPCC, MSE	PURE, COHFACE, UBFC-rPPG, MMSE-HR	HR	Reduce the noise interference from illumination variation and head movement
	2022	—	ResNet-18	No	Yes	Dlib to locate cheek area on generated mask datasets	10^–4^	—	Cross-entropy	Self-made mask dataset, COHFACE	RR	Judge whether the input signal is correct and further filter the measured outliers
	2022	—	ResNet-18	No	Yes	Dlib to detect face and KLT algorithm to track ROI	5 × 10^−4^	—	MSE, MAE	MAHNOB-HCI, VIPL-HR, UBFC-rPPG, MMSE-HR	HR	Preserve the feature vector of each frame in the video
	2023	—	ResNet101	No	No	Denoise and enhance for the captured actions by CNN	—	—	MSE	Aerobics training	HR, movement	Use the jump connection operation, and embed a lightweight module
	2023	WaveHRV	Complex-valued CNN	No	No	Came up with criteria based on biological restrictions and data analysis to filter out noisy ground truth data	—	—	MAE	Stroop, UBFC-rPPG, VIPL-HR, MAHNOB-HCI	HRV	Preprocess noisy contact-based PPG signals
	2023	GLISNet	2D convolution block and global-local interaction block	Hierarchical	Yes	—	2 × 10^−4^	—	NegPCC, MSE	PURE, UBFC-rPPG	HR	Extract and fuse pulse signals from multi-scale ROIs with lightweight computational load
RNN (+CNN) for BVP signal extraction	2019	LSTM-POS, CNN-POS	LSTM, CNN	No	No	Viola-Jones algorithm for face detection and HSV masking approach for skin segmentation	10^–3^	—	MSE	DEAP	HR	Aim to monitor people with profound intellectual and multiple disabilities
	2019	two-layer LSTM	LSTM	No	No	Dlib to detect facial landmarks	—	—	MSE	MMSE-HR	HR	Overcome the problem of insufficient HR public database
	2020	Meta-rPPG	2D ResNet, LSTM	No	Yes	Dlib to detect face and OpenCV to track	10^–3^	—	Ordinal regression loss	MAHNOB-HCI, UBFC-rPPG	HR	Propose a transductive meta-learner to cope with the unforeseeable distributional changes during deployment
	2020	RhythmNet	ResNet-18, RNN	No	Yes	SeetaFace to detect face and localize landmarks, skin segmentation to remove the non-face area	10^–3^	—	Smooth MAE	MAHNOB-HCI, MMSE-HR, VIPL-HR	HR	Consider the relationship of adjacent HR measurements from a video sequence; build a large-scale multi-modal HR database named VIPL-HR1
	2022	RobustPPG	Bi-LSTM	No	No	Weighting mask to remove facial hair or specular regions on non-Lambertian surfaces	—	—	MSE	PURE, RICE-Motion	HR	Filter the motion distortions in the rPPG signals
	2022	RGB-to-PPG DNN	U-Net, LSTM	No	No	Downsampling and interpolating for data augmentation	—	—	—	DEAP	PR	Need fewer data to extract reliable and faster PR
	2023	—	AlexNet, ResNet50V2, LSTM	No	No	Taking images of captured video streams	10^–2^	—	MSE, MAE	—	HR	Applied anywhere and anytime without needing equipment or special hardware
GAN for BVP signal extraction	2021	Dual-GAN	GAN, CNN	Channel	Yes	—	10^–4^	32	NegPCC, Cross-entropy	UBFC-rPPG, VIPL-HR, PURE	HR, HRV, RR	A jointly model both BVP predictor and noise distribution; obtain more robustness BVP representation against unseen noises
	2021	PulseGAN	Speech enhancement GAN	No	Time-frequency	Data augmentation to balance the reference HR distributions of training and testing databases	10^–3^	—	Adversarial, Spectrum, and Waveform loss	PURE, VIPL-HR, BSIPL-RPPG, UBFC-RPPG, MAHNOB-HCI	HR, IBI, HRV	The error losses defined in time and spectrum domains are both employed with the adversarial loss to enforce the model
	2023	PFDNet	GAN, CNN, LSTM	Self-attention	Yes	Viola-Jones algorithm for face detection, DRMF for location, KLT algorithm for tracking	10^–3^	16	MSE, Cross-entropy	COHFACE, PURE, VIPL-HR	HR, HRV	Discover the common features of VPPG and PPG pulse signals for atrial fibrillation detection
	2023	cbPPGGAN	CycleGAN	No	Time-frequency	—	10^–4^	80	Cycle consistency loss	UBFC-rPPG, BH-rPPG	HR, HRV	Employ cycle consistency loss with a time-frequency constraint to guide the generator in learning a robust mapping between two waveforms

Abbreviations: DL, deep learning; HR, heart rate; HRV, heart rate variability; RR, respiratory rate; ST, spatio-temporal; CNN, convolutional neural network; RNN, recurrent neural network; GAN, generative adversarial network; LSTM, long short-term memory; EVM, energy variance maximization; KLT, kanade lucas tomasi; DRMF, discriminative response map fitting; RICE, remote sensing image cloud removing; MAE, mean absolute error; MSE, mean squared error; NegPearson, negative Pearson correlation coefficient; PCC, pearson correlation coefficient; SNR, signal-to-noise ratio.

The interest in contactless or remote HR measurement has steadily grown in healthcare and sports applications. Contactless methods involve the utilization of a video camera and image processing algorithms. Due to rapid development in ML, DL methods have shown significant promise in improving the performance of conventional algorithms for contactless HR estimation. As large labeled open-source datasets are used to train these algorithms, high-quality and diverse datasets are crucial for proper benchmarking and analysis of different methods and the future development of more complex DL models and architectures. In the longer term, the continuous update and iteration of smartphones and the popularity of robots in public places will provide a stronger foundation for HR contactless monitoring (Siddiqui et al., 2016; Poh and Poh, 2017; Lee et al., 2022b).

## 5 Heart/pulse rate variability

Heart rate variability (HRV) refers to the change of interval time between continuous heartbeats, while pulse rate variability (PRV) refers to the change of pulse interval time in relation to the BVP signal, indicating the change of instantaneous PR/HR. Both HRV and PRV reflect the ability of the autonomic nervous system to maintain the balance of the internal environment. The difference between the two methods is that HRV is usually calculated by ECG, while PRV is obtained by PPG signal. The analysis of HRV and PRV is a useful tool for a comprehensive description of autonomic dynamics and can provide useful information about changes in vagus nerve activity (which can be used to monitor stress and mood changes) (Rajendra acharya et al., 2006).

### 5.1 Relevance between HRV and PRV

The HRV standard defines the HRV evaluation of long-term (LT; 24 h) and short-term (ST; 5 min) through time-domain, frequency-domain, and non-linear metrics (Malik et al., 1996). In recent years, to achieve the lowest possible power consumption and computing load, the HRV evaluation index of ultra-short term (UST; less than 5 min) has been proposed. By combining UST with wearable technology or smartphone applications, one can assess a person’s wellbeing (mood, stress, health) while being user-friendly (speed and comfort) (Nussinovitch et al., 2011; Munoz et al., 2015; Castaldo et al., 2019; Finžgar and Podržaj, 2020). Studies have shown that PRV can be used as an effective and accurate index for estimating HRV in healthy subjects at rest as this helps simplify the recording of the signals used in HRV assessment. However, under physical or mental stress, motion artifacts would lead to a decrease in the level of consistency between HRV and PRV, amongst which UST-HRV and ST-HRV may be more affected (Schäfer and Vagedes, 2013; Iozzia et al., 2016). It has been shown that it is possible to use rPPG signals to generate HRV information in subjects with autonomic nerve excitation. Moreover, the rPPG signal extracted by POS and CHROM methods is the most accurate in predicting autonomic dynamics (Van et al., 2023). In addition, the multiple simultaneously acquired BVP signals extracted by the ICA algorithm seem to be able to evaluate HRV reliably (Favilla et al., 2019). A PhysioCam system developed by Davila et al. (2017) extends the application scenario of PRV characterization of HRV based on the rPPG signal. Its performance is similar to that of standard signals (ECG and PPG) in three physiological conditions (rest, single deep breath, and continuous fast and shallow breathing). However, the balance of achieving user-friendly and accurate PRV assessment (consequently HRV) in patients with multiple comorbidities is still a difficult one to strike at this point.

### 5.2 Conventional methods for contactless HRV/PRV estimation

Unlike the evaluation of HR or PR, the measurement of HRV or PRV requires accurate peak detection of BVP signals and continuous extraction of PR and BVP signals. This usually has a higher noise level and lower temporal resolution than cPPG thus rendering contactless remote measurement of PRV to be more complex. To achieve accurate measurement of PRV, some CV researchers have tried to improve the performance of the camera. This often makes the process more complex, expensive, and not applicable in daily life (Sun et al., 2012; Mcduff et al., 2014; Mcduff et al., 2018). It seems to be a potential method to improve the algorithms, such as improving BVP peak recognition, improving time-domain resolution, magnifying the subtle changes of respiration and skin color, and combining face detection and tracking (Sun et al., 2012; Melchor Rodríguez and Ramos-Castro, 2018; Li et al., 2020; Pai et al., 2021; Yu et al., 2021).

Using the periodic variance maximization (PVM) method to extract the BVP signal on rPPG, and using the event-related two-window algorithm to improve BVP peak recognition, contactless and accurate PRV detection based on rPPG can be realized (Li et al., 2020). Interpolating can compensate for the negative effects of a low initial sample rate and improve time-domain resolution and PRV measurements, thus providing further strong support for the low-cost webcam-based rPPG technique (Sun et al., 2012). A method based on YCbCr chromatic aberration developed by Yu et al. (2021) magnifies the subtle changes of skin color to make it easier to identify, and realizes the continuous extraction of BVP signals, which breaks away from the limitation that conventional rPPG techniques only measure a single PR instead of the whole signal. Furthermore, Melchor Rodríguez and Ramos-Castro (2018) utilized the Viola-Jones face detection algorithm and Kanade-Lucas-Tomasi (KLT) tracking algorithm to process the video obtained by webcam, and achieve robust rPPG PRV analysis under small-range motion conditions. Still, this method does not take into account more extensive and complex motion types. Pai et al. (2021) have developed an HRVCam algorithm based on a frequency demodulation framework (a combination of a new automated adaptive bandpass filter and the discrete energy separation algorithm (DESA)) for subjects with large changes in respiration and skin color, which was used to estimate the instantaneous frequency of the rPPG signal, thus improving the accuracy of estimated time-domain HRV metrics. These improved algorithms have achieved good results on the datasets based on traditional low-cost cameras and may be suitable for the promotion of rPPG monitoring physiological signs. Table 2 summarizes these conventional rPPG signal extraction algorithms in contactless HRV/PRV estimation.

### 5.3 DL model for contactless PRV estimation

#### 5.3.1 End-to-end DL model

**FIGURE 4:**
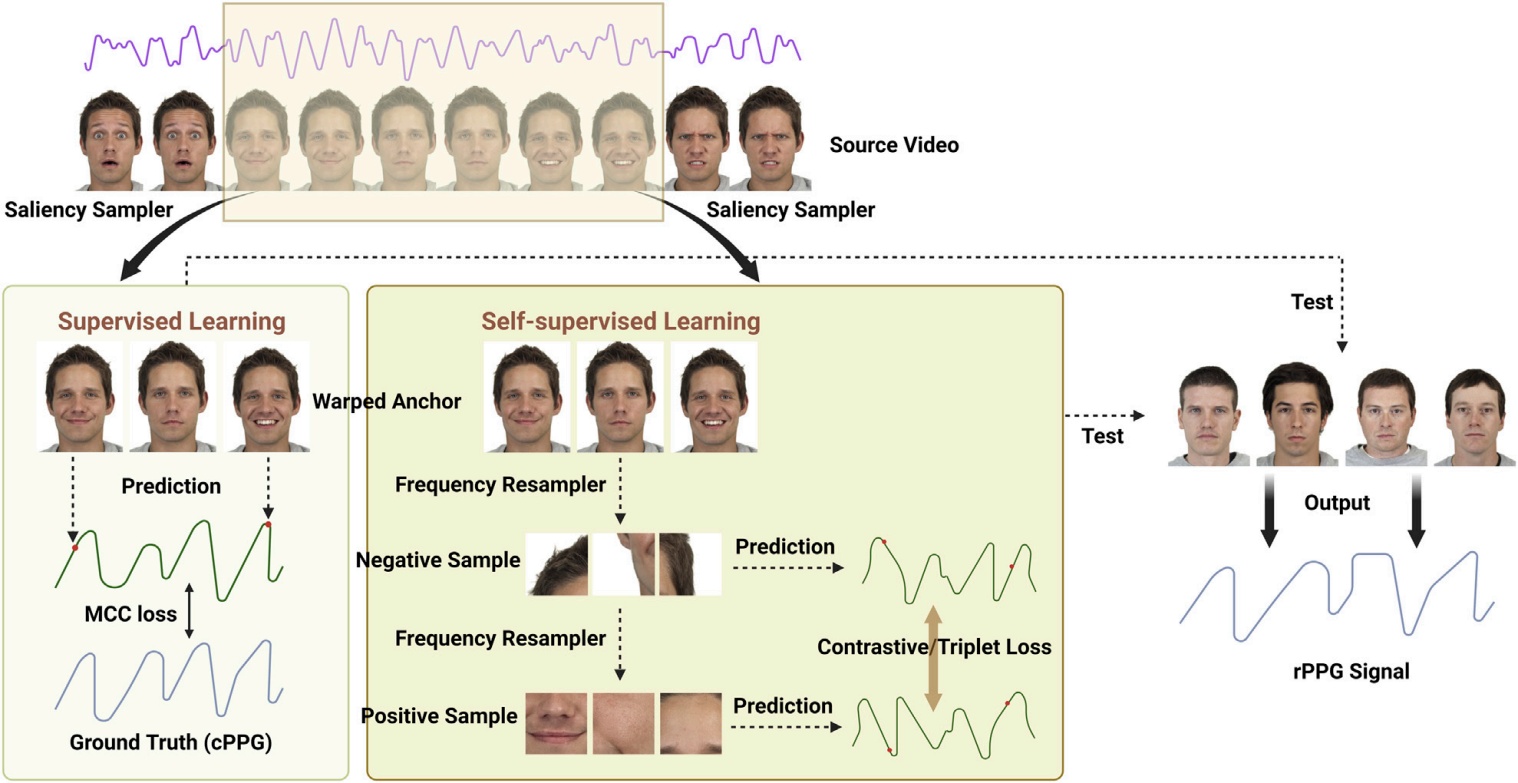
Supervised and self-supervised learning in rPPG signal prediction. A video clip is sampled from the source video first, then passed through the saliency sampler to generate the warped anchor. The anchor is passed through a PPG Estimator to get rPPG signal. If supervised training is employed, we employ a maximum cross-correlation (MCC) loss between the ground truth (cPPG) and predicted rPPG signal. If contrastive training is used, a random frequency ratio is sampled from a prior distribution. The warped clip is passed through the frequency resample to produce the negative sample, showing a subject with an artificially higher heart rate. This sample is passed through to produce the negative example PPG. The negative sample is again resampled with the inverse of random frequency ratio to produce a positive example PPG. Finally, the contrastive loss, multi-view triplet loss, is applied to the PPG samples, using a PSE MSE distance metric. The face images in the schematic diagram come from the Chicago Face Database (Ma et al., 2015).

The measurement of HRV/PRV is based on the accurate detection of HR/PR, and the DL model involved can be roughly divided into end-to-end and hybrid DL. PhysNet, an end-to-end ST network constructed by 3D CNN or 2D CNN + RNN, can accurately evaluate the measurement metrics characterizing HRV however is highly complex and time-consuming (Yu et al., 2019b). In addition, a 3D CNN architecture without skin segmentation or other preprocessing was developed to realize HRV measurement (Luguev et al., 2020). More recently, an efficient ST attention network (ESA-rPPGNet) was developed, which is composed of ESA (based on MobileNet v3), 3D shuffle attention, and gated recurrent unit (GRU) (Kuang et al., 2022). ESA-rPPGNet can recover high-quality rPPG signals to accurately locate the peak of each heartbeat, thus improving the accuracy of HRV analysis and reducing the time complexity of the network. However, these methods are trained in a supervised manner, where PPG signals are recorded synchronously with facial videos for supervision. A novel frequency-inspired self-supervised framework for facial video-based remote physiological measurement was proposed, which learns to optimize rPPG estimation from multiple augmented videos of different signal frequencies and across temporally neighboring videos of similar signal frequencies, while there is no demand for PPG signal originating from ground truth (Yue et al., 2023). It has three main stages: data augmentation (involving a 3D Convolution layer, 3D Res-blocks, and Bi-LSTM), signal extraction (based on 3D ResNet-10), and network optimization. Its performance was better than most advanced self-supervised methods and equivalent to the most advanced supervised methods in HR, HRV, and RR estimation. Figure 4 shows the difference between supervised and self-supervised learning in rPPG signal prediction. Table 3 summarizes the application of end-to-end DL methods in contactless HRV estimation.

#### 5.3.2 Hybrid DL model

Wavelet scattering transform, a complex-valued CNN model, can denoise an extracted rPPG signal (Odinaev et al., 2023). Combined with adaptive bandpass filtering and inter-beat-interval (IBI) analysis, the contactless detection of HRV can be achieved. This transformation has been verified on different public datasets with satisfactory results. The proposed PulseGAN framework employs a combination of waveform, spectrum, and adversarial losses to enable extraction of high-quality rPPG pulse waveforms from rough input signals obtained by conventional methods (e.g., CHROM) to infer reliable cardiac features (e.g., HRV) (Song et al., 2021). In addition, the cbPPGGAN predicts a more realistic pulse waveform and a more accurate HRV estimation (Yang et al., 2023).

Cardiovascular disease is one of the most common diseases, and HRV may be a valuable indicator for predicting sudden cardiac death and arrhythmias. With increasing societal pressures, the youth will increasingly experience mental health and emotional stressors. As a physiological index reflecting stress and emotional changes, HRV monitoring helps evaluate the mental health of adolescents and prompts early intervention from psychiatrists. Real-time monitoring of HRV in various scenarios helps detect the occurrence of cardiovascular diseases and mental diseases, thus providing an early detection mechanism for a variety of global health problems.

## 6 Blood pressure

In the field of remote healthcare, non-invasive continuous BP measurement has become a growing topic. Classic non-invasive BP measurement techniques can obtain spontaneous systolic blood pressure (SBP) and diastolic blood pressure (DBP) at a point in time while invasive BP measurement techniques provide continuous BP monitoring. These techniques are however not suitable for long-term monitoring due to discomfort and are generally used in intensive care units. With the development of telemedicine, the demand for non-invasive continuous BP monitoring will continue to increase.

### 6.1 rPPG for contactless BP estimation

The research shows that the pulse transit time (PTT) determined by BP can be expressed not only by the time lag between the R wave of ECG and a subsequent pulse wave but also by the time lag between two PPG’s measured at different body locations. The principle of measuring BP by rPPG technology is based on its recognition of PTT (Geddes et al., 1981; Nitzan et al., 2002; Mukkamala et al., 2015; Sugita et al., 2015; Jeong and Finkelstein, 2016; Secerbegovic et al., 2016; Zhou et al., 2019; Fan et al., 2020). In addition to PTT-based methods, cuffless BP measurements are implemented by pulse arrival time (PAT, which requires an ECG sensor and a PPG sensor), pulse wave velocity (PWV, which requires two PPG sensors), and pulse wave analysis (PWA, which requires a PPG sensor) (Mccombie et al., 2006; Kim et al., 2015; Liu et al., 2017; El-Hajj and Kyriacou, 2020). On devices, it seems feasible for near-infrared cameras and smartphones to obtain rPPG signals that can characterize PTT. The accuracy is however affected by noise and motion artifacts (Krejcar et al., 2009; Chandrasekaran et al., 2013; Visvanathan et al., 2013). The development and optimization of algorithms is an effective means to achieve accurate contactless BP measurement. The development of AI represented by DL has brought revolutionary changes to contactless BP measurement.

### 6.2 DL model for contactless BP estimation

Research shows that artificial neural networks (ANN) can extract BP signals from face and finger videos (Lamonaca et al., 2013; Gonzalez et al., 2018; Luo et al., 2019). BP estimation algorithm based on DNN is one of the main research directions of continuous non-invasive BP monitoring by feeding features or waveforms to a neural network. Compared with the conventional ML-based measurement methods, DL models have a stronger ability to learn high-dimensional features and a better fit for complex nonlinear relationships.

#### 6.2.1 Single CNN model

Using only one CNN model in one phase to realize BP estimation is defined as a single CNN model. The abilities of various DL algorithms (RhythmNet, GoogleNet, CNN with network regularization and attention module, ResNet50, ResNet18, VGG16 with BN layer, Small-rPGGNET, lightweight VGG16) to deal with RGB green channel 1D signal are compared (Xing et al., 2023). Among them, the simplified lightweight VGG16 network has the advantages of fewer network layers and rapid training convergence. It can achieve its best performance of BP estimation from facial videos. The DL algorithm based on the U-Net structure developed by Bousefsaf et al. (2021) can convert the rPPG signal acquired by wavelet transform into the cPPG signal, and successfully estimate BP from the cPPG signal. However, the videos involved in this study were captured by a fast camera, whose signals do not completely reflect those constituted from frames delivered by conventional cameras or webcams. Lin et al. (2023) proposed a method based on video magnification and DL which reduces the influence of interferences from human skin characteristics, breathing, and the external environment by extracting dual-path time series from facial video. This resulted in a highly precise estimation of vital signs. In this model, although the learning-based video motion magnification (VMM) algorithm can achieve the best accuracy, EVAM can better balance the running time and accuracy, while the small two-stage CNN algorithm can predict BP by extracting features from stable time series rather than the whole image, thus maintaining the effectiveness of training under limited samples.

#### 6.2.2 Hybrid CNN model

Using different CNN models in one phase or different phases is defined as a hybrid CNN model. Iuchi et al. (2022) proposed a CNN architecture based on ResNet and CBAM, which established the relationship model between spatial information of facial pulse waves and BP, while the pattern of pulse contour-wise contribution pattern reflects the relationship between percussion wave and dicrotic wave. It was able to achieve its purpose of extracting continuous BP from RGB video. Wu et al. (2022) proposed three customized CNNs (Feature- Based Networks, Signal-Based Networks, and Feature-Signal-Combined Networks) based on residual blocks from ResNet, which use physiological indicators (including HR, HRV, BMI, and PTT) and multi-channel rPPG signals as model inputs. These calibration-free characteristics greatly improve the convenience, expand the application scope, and are widely verified in a large number of datasets of real patients who require BP monitoring. However, a single training dataset, long BP measurement time, and video resolution are vital factors that limit the generalization ability of the model. Joung et al. (2023) developed the PPG2BP-Net [(comprises a comparative paired 1D CNNs, one multi-layer perceptron (MLP), and one fully connected layer (FCL)] based on the large sample database with highly varying intrasubject BP which enabled the measurement of varying BP accurately in new daily users as the proposed subject-independent approach is regenerative for a new subject.

#### 6.2.3 Hybrid DL model

Using CNN and DNN models at the same time to realize BP estimation is defined as a hybrid DL model. Hybrid DL models, including CNN, LSTM, and FCL, developed by Hamoud et al. (2023), can predict BP from images of ROI cropped from each frame of the video with just a smartphone. While this hybrid model establishes a link between BP and RR, there is a lack of datasets including populations with skin color changes and hypertension for verification. Cheng et al. (2023) proposed a multi-stage DL model based on rPPG signal, which combines CNN and bidirectional GRU (a variant network of LSTM) neural networks to automatically extract different morphological features of SBP and DBP waveforms. The proposed bidirectional GRU can establish the feature association between future information and past information, which solves the time series data features that are forgotten, thus reducing workload and improving the accuracy of BP measurement. Wu et al. (2023) proposed a multi-model structure, including face rPPG signal extraction (using multi-task cascade CNN), time difference feature extraction, the DL model architecture, model selection with subject information (considering the influence of BMI and age on BP), and synthetic data generation with InfoGAN (generates specified data by learning mutual information between latent noise and observations) to eliminate overfitting by the DL model and compensate for the lack of data. It was able to achieve good BP estimation on multiple rPPG datasets. Table 5 summarizes the application of the DL model in contactless BP estimation.

**TABLE 5:** Application of DL model in contactless BP estimation.

Publication year	DNN type	Backbone network	Data preprocessing	Learning rate	Batch size	Loss function	Datasets	BP computation methods	Purpose
2021	CNN	U-Net	Dlib to locate forehead	10^–4^	16	MSE	Self-constructed	PWA	Convert iPPG signals measured using a camera into cPPG signals measured by contact sensors
2022	CNN	ResNet, CBAM	ICA to separate components then remove shading	10^–2^	256	MSE	Self-constructed	PWA	Estimate continuous BP based on spatial information of a pulse-wave as a function of time
2022	CNN	ResNet	RetinaFace and Dlib for location	5 × 10^−2^	—	MSE	Morph-II, Self-constructed	PTT	Evaluate algorithms with large enough datasets to be effective
2023	CNN	—	Face and neck respectively are extracted, tracked, and magnified, then PCA for BSS	—	—	RMSE, MAPE	Self-constructed	PTT	Solve the common problems of rPPG including weak extracted signals, body movements, and generalization with limited data resources
2023	CNN	ResNet50, ResNet18, GoogleNet, VGG16	Dlib to track and annotate five regions based on TCM face diagnosis (heart, liver, spleen, lung, and kidney)	—	—	MAE	Self-constructed	PWA	Screen the best performed prediction model; investigated the impact of face reflex regions selection on BP prediction model
2023	CNN	1D CNN, MLP, FCL	Data elimination, downsampling, segmentation, normalization, and balancing	10^–4^	64	—	VitalDB	PWA	Realize subject-independent and highly varying BP estimation
2023	CNN, RNN	Xception, DenseNet121, VGG16, Resnet50V2, InceptionV3, EfficientNet, FCL, LSTM	3D Total Solution 3DDFA framework to segment the faces and extract the forehead, right cheek, and left cheek regions	10^–4^	—	MSE	V4V, Self-constructed	PWA	Present an innovative, inexpensive, and time-efficient method to estimate blood pressure using only a smartphone camera
2023	CNN, GAN	Multitask cascade CNN, InfoGAN	MTCNN to locate five facial landmarks, skin detector to filter out non-skin parts, upsample and interpolate to enhance the physiological information	2 × 10^−3^	—	—	rPPG, Dynamic, TVGH, Self-constructed	PWA	Prevent overfitting and compensate for the lack of data
2023	CNN, RNN	1D CNN, BiGRU	Wavelet transforme to remove the baseline drift, Butterworth bandpass filter to remove the high-frequency noise	10^–3^	32	CCE	Self-constructed	PWA	Consider the physiological relationship between diastole and systole

Abbreviations: DL, deep learning; BP, blood pressure; DNN, deep neural network; CNN, convolutional neural network; RNN, recurrent neural network; GAN, generative adversarial network; LSTM, long short-term memory; PWA, pulse wave analysis; PTT, pulse transit time; CBAM, convolutional block attention module; MLP, multilayer perceptron; FCL, fully connected layer; BiGRU, bidirectional gated recurrent unit; CCE, categorical cross-entropy; MAE, mean absolute error; MSE, mean squared error; RMSE, root mean squared error; MAPE, mean absolute percentage error; TVGH, Taipei Veterans General Hospital.

Hypertension is the leading cause of death worldwide and a key risk factor for many serious diseases, including cardiovascular diseases such as stroke and heart failure. BP is a major vital sign and must be monitored regularly for early detection, prevention, and treatment of cardiovascular disease. Conventional BP measurement techniques (invasive or cuff-based) are impractical, intermittent, and uncomfortable for patients. The method based on rPPG can realize the contactless monitoring of BP with improved patient comfort and mobility. CV-based methods can fully combine the advantages of computer algorithms and can extract key information characterizing BP from simple images or videos. With the development of DL, an exciting new field for contactless and continuous BP monitoring based on rPPG has been opened up. This will have a significant and transformative impact on monitoring the vital signs of patients, particularly those with high cardiovascular risk factors or diseases. It is encouraging to see a great amount of interest from both researchers and industry alike. While there are still challenges ahead, the continuous and relentless momentum of research provides hope for future PPG-based non-invasive, cuff-less, and continuous BP monitoring devices in the near future.

## 7 Limitations, prospects, and conclusion

There is already a tremendous amount of real-world applications for CV, and the technology is still young. Besides Healthcare, Autonomous vehicles, Google Translate app, Facial recognition, Real-time sports tracking, Agriculture, and Manufacturing are inseparable from the popularization of CV. As humans and machines continue to partner, the human workforce will be freed up to focus on higher-value tasks because the machines will automate processes that rely on image recognition. However, the popularity of AI will bring some problems. Data privacy issues are particularly common and prominent in the field of CV. With the open-source of a large number of datasets such as COHFACE, MAHNOB, and PURE, extensive videos and photographs containing face or identity information are disclosed. In the context of big data, in addition to adopting technical measures including anonymization, differential privacy, local differential privacy, and homomorphic encryption, strengthening data management is a another vital means to balance medical data sharing and privacy security. However, the application of these data privacy protection technologies needs to consider their efficiency and impact on data availability. The security management of medical and health data involves many departments, including medical institutions, AI suppliers, medical information management departments, etc. They are responsible for data collection, mining, storage, application and transmission. Therefore, the relevant departments are supposed to establish a security management system, series standard operating procedures, and a credible network security environment, strengthen supervision, reasonably utilize medical and health data in accordance with regulations, strictly standardize data use rights and data access control to protect data privacy and data security. Besides the concern about data privacy, another factor that influences remote contactless physiological monitoring must be considered, that is, the poor generalization of current task-specific algorithms, which causes weak accessibility for underserved populations. Generalist medical AI (GMAI), as a new concept proposed in recent years, can perform a variety of tasks using minimal or no task-specific labeled data (Moor et al., 2023). However, the development of GMAI usually founds on massive datasets, which brings about privacy issues. Therefore, when applying GMAI to the field of CV, we must consider the ethical issues and security risks involved, so that it can develop in a direction beneficial to accessible remote physiological monitoring for human health.

This paper aims to provide an in-depth and comprehensive literature review of the existing and proposed Artificial Intelligence methods with a focus on computer vision and deep learning in contactless physiological monitoring. Contactless physiological monitoring techniques based on images or video represented by rPPG have been applied in the evaluation of microcirculation perfusion, respiratory rate, oxygen saturation, heart rate, heart rate variability, and blood pressure while overcoming the limitations of conventional contact physiological measurements. The development of deep learning has injected new vitality into this field. Alongside continuous optimization of traditional algorithms, the gradual maturity of deep learning algorithms, and the miniaturization of imaging equipment, there is hope that these advancements will contribute greatly to comfortable, portable, and cost-effective remote healthcare services in the near future.
